# Phemenology of Filling, Investigation of Growth Kinetics and Electronic Properties for Applications of Filled Single-Walled Carbon Nanotubes

**DOI:** 10.3390/nano13020314

**Published:** 2023-01-12

**Authors:** Marianna V. Kharlamova, Christian Kramberger

**Affiliations:** 1 Centre for Advanced Materials Application (CEMEA), Slovak Academy of Sciences, Dúbravská cesta 5807/9, 845 11 Bratislava, Slovakia; 2 Faculty of Physics, University of Vienna, Boltzmanngasse 5, 1090 Vienna, Austria

**Keywords:** carbon nanotube, filling, kinetics, electronic properties, Raman spectroscopy, near edge X-ray absorption, fine structure spectroscopy, photoemission spectroscopy, optical absorption spectroscopy

## Abstract

This review discusses the phemenology of filling, the investigation of kinetics, and the electronic properties for applications of filled single-walled carbon nanotubes (SWCNTs), and summarizes five main achievements that were obtained in processing the spectroscopic data of SWCNTs filled with metal halogenide, metal chalcogenide, metal and metallocenes. First, the methods of processing kinetic data were developed to reveal precise trends in growth rates and activation energies of the growth of SWCNTs. Second, the metal-dependence of kinetics was revealed. Third, metallicity-sorted (metallic and semiconducting) SWCNTs were filled with a range of substances and the electronic properties were investigated. Fourth, new approaches to processing the data of spectroscopic investigations of filled SWCNTs were developed, which allowed more reliable and precise analysis of the experimental results. Fifth, the correlation between the physical and chemical properties of encapsulated substances and the electronic properties of SWCNTs were elucidated. These points are highlighted in the review.

## 1. Introduction

**Figure 1:**
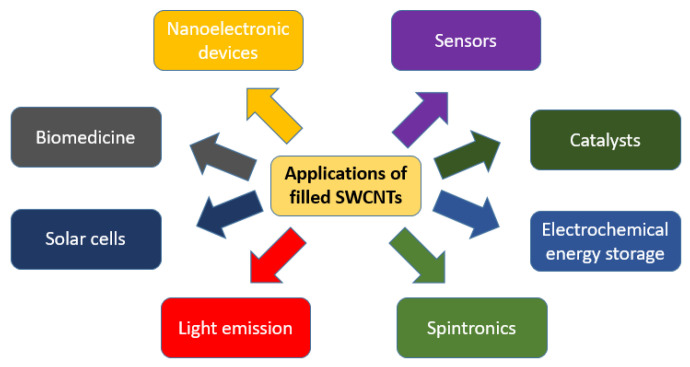
Applications of filled SWCNTs.

Single-walled carbon nanotubes (SWCNTs) are promising materials for a wide range of applications, such as molecular electronics, nanocomposites, solar cells, sensors, and electrochemistry lithium-ion batteries (Figure 1) [1,2,3,4,5,6,7,8,9].

SWCNTs are modified in different ways to perform the functionalization of the inner, outer surface and insert substances in inner channels, i.e., perform filling. These methods include covalent functionalization of the outer surface, noncovalent functionalization, substitution of atoms in the walls of SWCNTs, electrochemical doping, filling and nanochemical reactions within the nanotube channels [10]. The growth rates and activation energies of the growth of carbon nanotubes inside SWCNTs are calculated [11,12]. The kinetics of growth of the inner tubes are studied in detail by the annealing of metallocene-filled SWCNTs with a resolution of several minutes. It has been shown that nickel catalyst leads to higher growth rates at lower temperatures than cobalt catalyst. The activation energies are different for the two metals. Understanding the growth mechanism of SWCNTs leads to a better understanding of growth and electronic properties. The electronic properties of filled SWCNTs are studied mainly by Raman spectroscopy, near-edge X-ray absorption fine structure spectroscopy (NEXAFS), photoemission spectroscopy (PES) and optical absorption spectroscopy (OAS) [13,14,15,16,17,18,19,20,21,22,23,24,25,26,27,28,29,30,31,32,33,34,35,36,37,38,39,40,41,42,43,44]. Raman spectroscopy shows that there is a charge transfer in nanotubes filled with 3d, 4d, 4f, 5s and 6p-metal halogenides. We observed changes in the spectra shapes and shifts of peaks. NEXAFS reveals the formation of local chemical bonds. Photoemission spectra reveal shifts in the peaks. i.e., direct confirmation of doping with a charge transfer. The optical absorption spectroscopy confirms the charge transfer. These studies, which have been performed for almost two decades, are very important. They can lead to many excellent applications, in many different countries, including Russia. 

**Figure 2:**
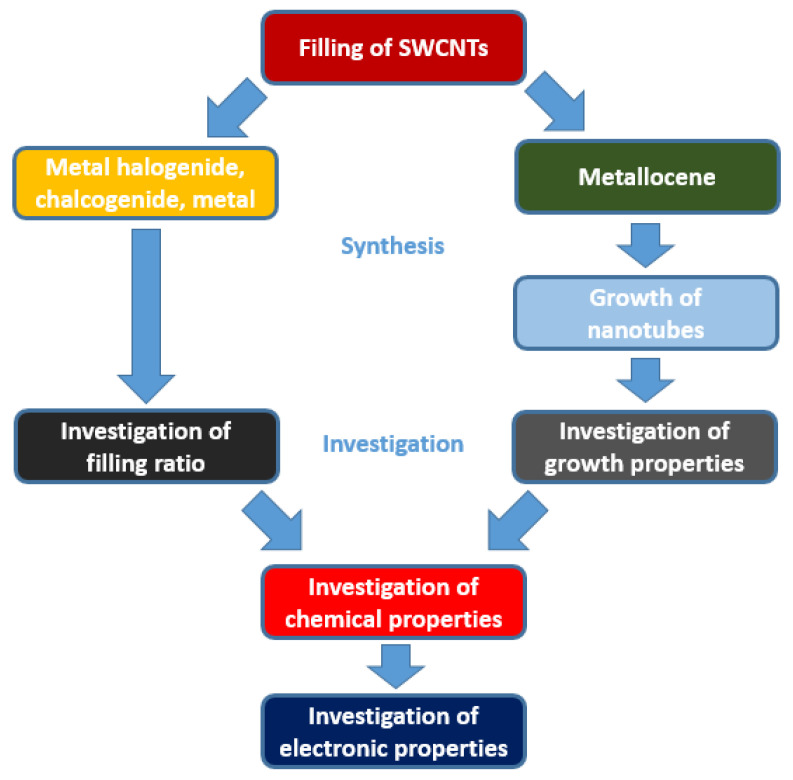
The schematics of filling of SWCNTs with metal halogenides, metal chalcogenides, metals, metallocenes, and investigation of growth properties, chemical properties and electronic properties.

In this review we discuss the phemenology of filling, the investigation of growth kinetics, and the electronic properties for the application of SWCNTs (Figure 2). 

The SWCNTs were filled with molecules [45,46,47,48,49,50,51,52,53,54,55,56,57,58,59,60,61,62,63,64,65,66,67,68,69,70,71,72,73,74,75,76,77,78,79,80,81,82,83,84,85,86,87,88,89,90,91,92,93,94,95,96,97,98,99,100,101,102,103,104,105,106,107,108,109,110,111,112,113,114,115,116,117,118,119], metals and nonmetals, such as Ag, Cu, P, As, and I [120,121,122,123,124,125,126,127,128,129,130,131,132,133,134,135,136,137,138,139,140,141,142,143,144,145,146,147,148,149,150,151,152,153], metal halogenides, such as MHal (M=Ag, Cu, Hal=Cl, Br, I), MHal_2_ (M=Fe, Co, Ni, Mn, Zn, Cd, Pb, Hg, Hal=Cl, Br, I), and MHal_3_ (M=Pr, Tb, Tm, Lu, Hal=Cl, Br, I) [154,155,156,157,158,159,160,161,162,163,164,165,166,167,168,169,170,171,172,173,174,175,176,177,178,179,180,181,182,183,184,185,186,187,188,189,190,191,192,193,194,195,196,197,198,199,200,201,202,203,204,205,206,207], and metal chalcogenides, such as MX (M=Ga, Sn, X=S, Se, Te), and M_2×3_ (M=Bi, X=Se, Te) [208,209,210,211,212,213,214]. They are filled inside SWCNTs by the gas or melt methods; the synthesis process involves thermal treatment with cooling, and an investigation with spectroscopic techniques for applications. 

In Section 2 of this review, we consider the kinetics of growth of carbon nanotubes, and trends in the metal-dependence of growth rates and activation energies. In Section 3, we carry out an investigation into the doping effects in metallicity-sorted SWCNTs filled with inorganic compounds. In Section 4, we produce a comparison of the doping effect of different inorganic compounds on SWCNTs. Section 5 comprises a comparison of the doping effect of inorganic compounds on different diameter SWCNTs. In Section 6, a discussion of the influence of different encapsulated substances on the electronic properties of SWCNTs is performed. 

## 2. Studies on Filled SWCNTs

Molecules have been encapsulated inside SWCNTs in 74 papers [45,46,47,48,49,50,51,52,53,54,55,56,57,58,59,60,61,62,63,64,65,66,67,68,69,70,71,72,73,74,75,76,77,78,79,80,81,82,83,84,85,86,87,88,89,90,91,92,93,94,95,96,97,98,99,100,101,102,103,104,105,106,107,108,109,110,111,112,113,114,115,116,117,118,119], and the filled SWCNTs have been investigated for applications in spintronics, construction material, magnetic storage, and magnetic recording. These applications were reviewed by us recently [214]. They were also reviewed by the groups of Jeremy Sloan [215], Marc Monthioux [216], and Ferenc Simon [217]. The following studies are available:

- The filling process of fullerenes and derivatives, as well as applications were studied by Hiromichi Kataura, Kazu Suenaga, Sumio Iijima, and Hisanori Shinohara [49,51,52,58,70,71,72,75,76,79,80,116].
- Molecule-filled SWCNTs were synthesized for the investigation of growth kinetics of carbon nanotubes. The fullerenes coalesce and form an inner nanotube. The mechanism was studied by our group [218].
- The synthesis process and catalysis properties of endohedral fullerene-filled SWCNTs were studied by Andrey Khlobystov and Thomas Chamberlain [48,53,54,55,81,83,84,85,86,112].
- The filling process of SWCNTs with other molecules was analyzed by Ákos Botos and Katalin Kamaros [46].
- The carbon nanotubes were also filled with molecules other than fullerenes, e.g., metallocenes, by Hidetsugu Shiozawa [59,95,96,97,98,109,110,111] and Marianna Kharlamova [105,106,107,108]. The growth properties of molecule-filled SWCNTs were reviewed in Ref. [219] where the growth mechanisms of carbon nanotubes were also discussed extensively.

Metals and nonmetals have been encapsulated inside SWCNTs in 33 papers [120,121,122,123,124,125,126,127,128,129,130,131,132,133,134,135,136,137,138,139,140,141,142,143,144,145,146,147,148,149,150,151,152,153]. These papers are dedicated to the filling of SWCNTs with iodine, phosphorous, arsenic, sulfur, selenium, silver, copper, and europium.

- Extensive studies of the microstructure of filled SWCNTs have been performed.
- The filling processes of SWCNTs have been analyzed, and the procedures have been optimized for the filling.
- The electronic properties have been investigated by spectroscopy.

Among Russian scientists, Elena Obraztsova [123], Alexander Okotrub [129], and Marianna Kharlamova [141,142,147] filled SWCNTs with simple substances. 

Metal halogenides and metal chalcogenides were filled inside SWCNTs in 60 papers [154,155,156,157,158,159,160,161,162,163,164,165,166,167,168,169,170,171,172,173,174,175,176,177,178,179,180,181,182,183,184,185,186,187,188,189,190,191,192,193,194,195,196,197,198,199,200,201,202,203,204,205,206,207,208,209,210,211,212,213,214]. They are listed above. Here, we present an overview of the novelties in the investigation of the kinetics and electronic properties of filled SWCNTs. The following was achieved [10].

- The methods of processing of the kinetic data were developed to reveal precise trends in growth rates and activation energies of the growth of SWCNTs.
- The metal-dependence of kinetics was revealed.
- Metallicity-sorted (metallic and semiconducting) SWCNTs were filled with a range of substances. Indeed, only metallicity-mixed SWCNTs have previously been used for the filling [10]. The filling of metallicity-sorted SWCNTs allowed for the unambiguous assessment of the influence of encapsulated substances on the electronic properties of SWCNTs.
- New approaches to processing the data of spectroscopic investigations of filled SWCNTs were developed, which allowed for a more reliable and precise analysis of the experimental results and for the drawing of clear conclusions about the influence of different fillers on the electronic properties of SWCNTs.
- The correlation between the physical and chemical properties of encapsulated substances and their influence on the electronic properties of metallicity-sorted and mixed SWCNTs with different diameters was elucidated.

## 3. Kinetics of Growth of SWCNTs

The growth kinetics of SWCNTs are important, because it allows control of the synthesis process of carbon nanotubes. The parameters, such as the growth rates and activation energies of the growth of SWCNTs, allow us to choose synthesis conditions that lead to the highest yield of SWCNTs. The investigation of the growth mechanism of carbon nanotubes allows for the determining of the growth model and appropriate mathematical model for describing the experimental growth. 

In Refs. [11,12], the kinetics of growth of carbon nanotubes inside nickelocene- and cobaltocene-filled SWCNTs were investigated. The synthesis was performed from the gas phase, followed by an investigation into the atomic structure, growth properties, chemical properties and electronic properties for applications. 

**Figure 3:**
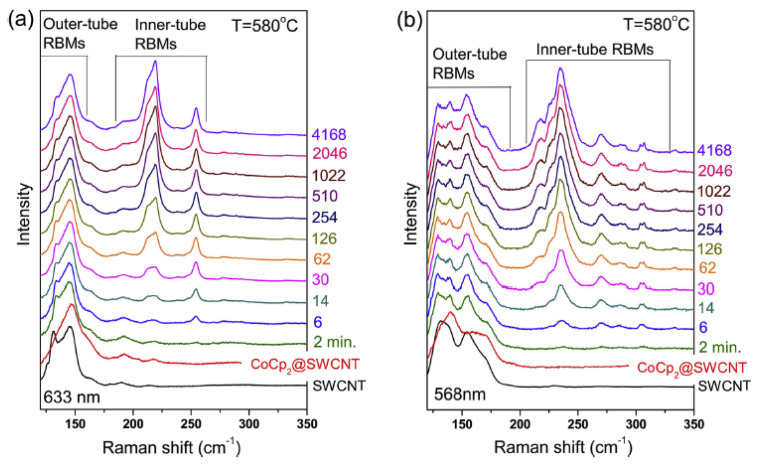
The Raman spectra of cobaltocene-filled SWCNTs annealed at temperature of 580 °C for 2–4168 min, acquired at laser wavelength of 633 nm (**a**) and 568 nm (**b**). Reprinted from M. V. Kharlamova et al. Chiral vector and metal catalyst-dependent growth kinetics of single-wall carbon nanotubes, Carbon, 2018, V. 133, pp. 283–292, Copyright 2018, with permission from Elsevier [11].

Figure 3 shows the Raman spectroscopy data of cobaltocene-filled SWCNTs annealed at a temperature of 580 °C for 2–4168 min, acquired at laser wavelength of 633 nm and 568 nm [11]. The spectra demonstrate the increase in intensity of peaks of the inner tube at 175–325 cm^−1^, which corresponds to a growth of inner SWCNTs inside filled SWCNTs. 

**Figure 4:**
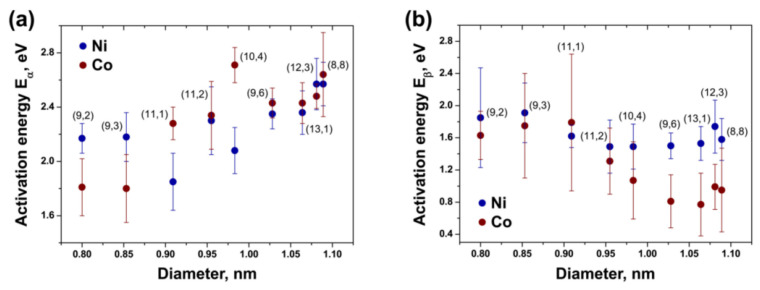
The dependence of activation energy E_α_ (**a**) and E_β_ (**b**) of growth of inner nanotubes on their diameter. Reproduced from [215]. Copyright 2021 by the authors. Licensee MDPI, Basel, Switzerland. This article is an open access article distributed under the terms and conditions of the Creative Commons Attribution (CC BY) license.

The growth rates and activation energies of the growth of inner SWCNTs were calculated for cobaltocene- and nickelocene-filled SWCNTs. Figure 4 compares the activation energies of the growth of nanotubes on cobalt and nickel clusters, formed upon the heating of metallocene-filled SWCNTs [220]. The activation energies E_α_ and E_β_ of an initial fast and subsequent slow growth on the purely metallic Ni and Co particles equal ~1.5–1.9 eV and ~0.8–1.8 eV, respectively. For large tubes with a diameter above ~0.95 nm on the Ni catalyst, they are significantly larger than on the Co catalyst, and the values of the smaller tubes are equal. The difference in the Ni and Co is due to different diffusion rates. 

## 4. Investigation of Doping Effects in Metallicity-Sorted SWCNTs Filled with Inorganic Compounds

**Figure 5:**
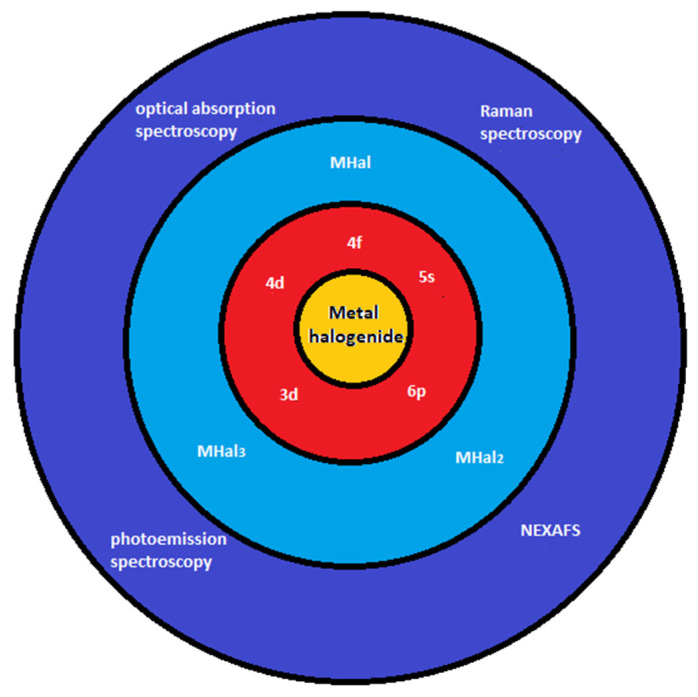
The schematic of metal halogenide-filling and investigation of SWCNTs.

Recently, new reports on the filling and investigation of metallicity-mixed and metallicity-sorted nanotubes were published (Figure 5). 

Table 1 summarizes the encapsulated inorganic compounds, diameter and conductivity types of the host SWCNTs, methods of the investigation of the electronic properties, types of doping and the observed Fermi level shifts of SWCNTs. 

The electronic properties of SWCNTs filled with manganese halogenides [175], iron halogenides [170], cobalt halogenides [190], nickel halogenides [167], zink halogenides, [169], cadmium halogenides [173], luthetium halogenides [184], mercury halogenies [172], and lead halogenides [177] were studied by Raman spectroscopy, near-edge X-ray absorption fine structure spectroscopy, photoemission spectroscopy, and optical absorption spectroscopy and p-doping with the Fermi level shift of −0.05–0.5 eV was revealed. 

**Figure 6:**
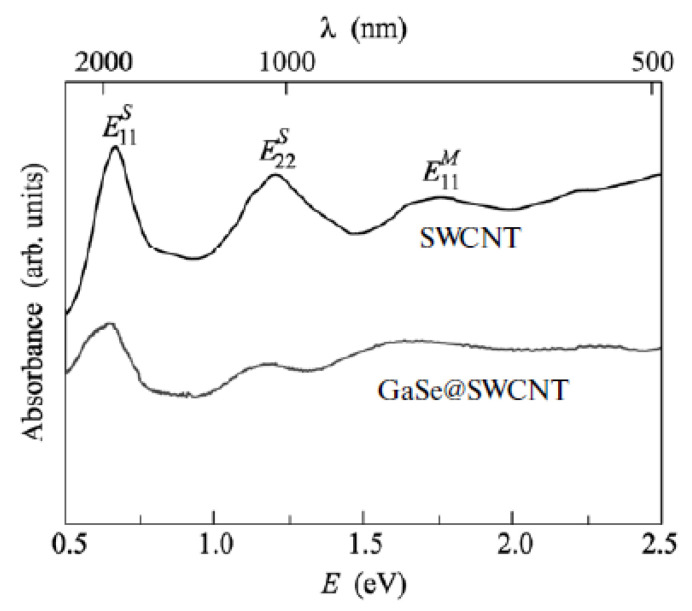
The OAS spectra of the pristine, and GaSe filled SWCNTs. The peaks are denoted. Reprinted from Kharlamova M.V. Novel approach to tailoring the electronic properties of single-walled carbon nanotubes by the encapsulation of high-melting gallium selenide using a single-step process. JETP Letters. V.98. N.5. P.272–277, 2013, Springer Nature [212].

OAS was applied to investigate the direction of the charge transfer in filled SWCNTs in Refs. [155,156,167,169,170,173,182,190,207]. The OAS spectra demonstrated the suppression of the peaks of Van Hove singularities. This was attributed to the doping of SWCNTs with a lowering or increasing of the Fermi level of nanotubes. However, the direction of the charge transfer cannot be deduced from the OAS data (Figure 6) [212]. 

**Figure 7:**
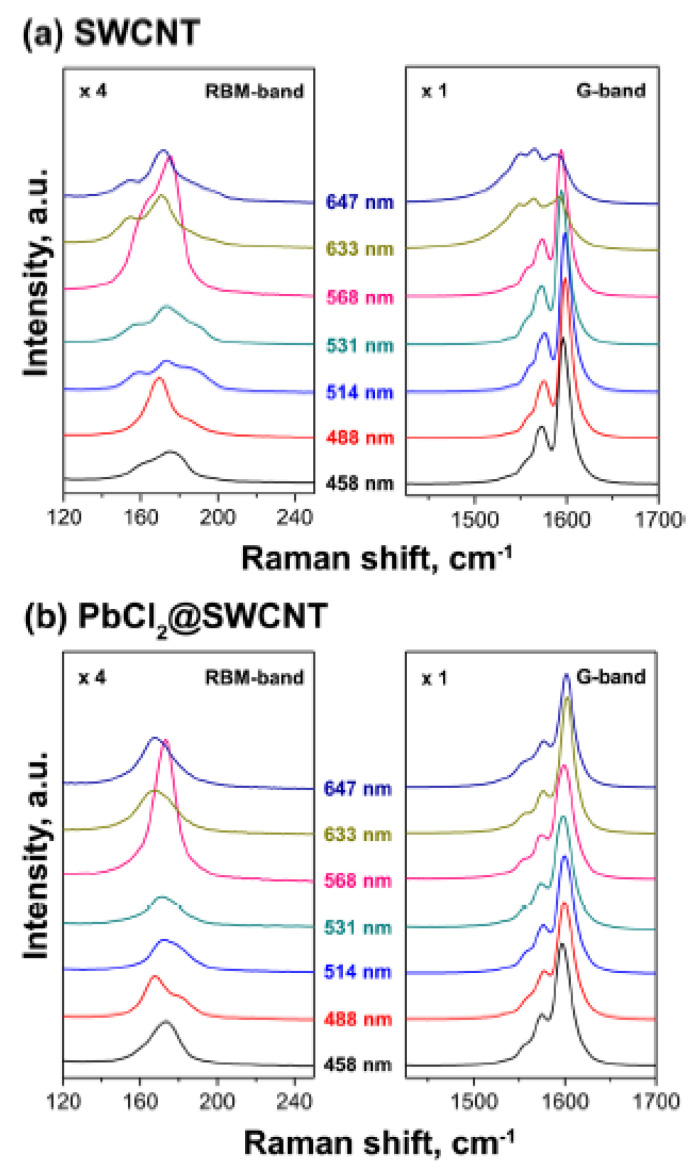
The Raman spectra of the pristine SWCNTs, and PbCl_2_—filled SWCNTs acquired at different laser excitation wavelengths. Reprinted from Kharlamova M.V., Kramberger C., Rudatis P., Pichler T., Eder D. Revealing the doping effect of encapsulated lead halogenides on single-walled carbon nanotubes. Appl. Phys. A. 2019. V.125. N.5. article number 320, Springer Nature [177].

Raman spectroscopy was also used to study the electronic properties of filled SWCNTs in Refs. [155,156,163,164,167,169,170,172,173,175,177,182,183,184,190,200,207,211]. Raman spectra revealed the changes in the radial breathing mode and G-mode, such as shifts in the peaks and modification of the band profile. This testifies to the doping of SWCNTs by encapsulated compounds (Figure 7) [177]. 

**Figure 8:**
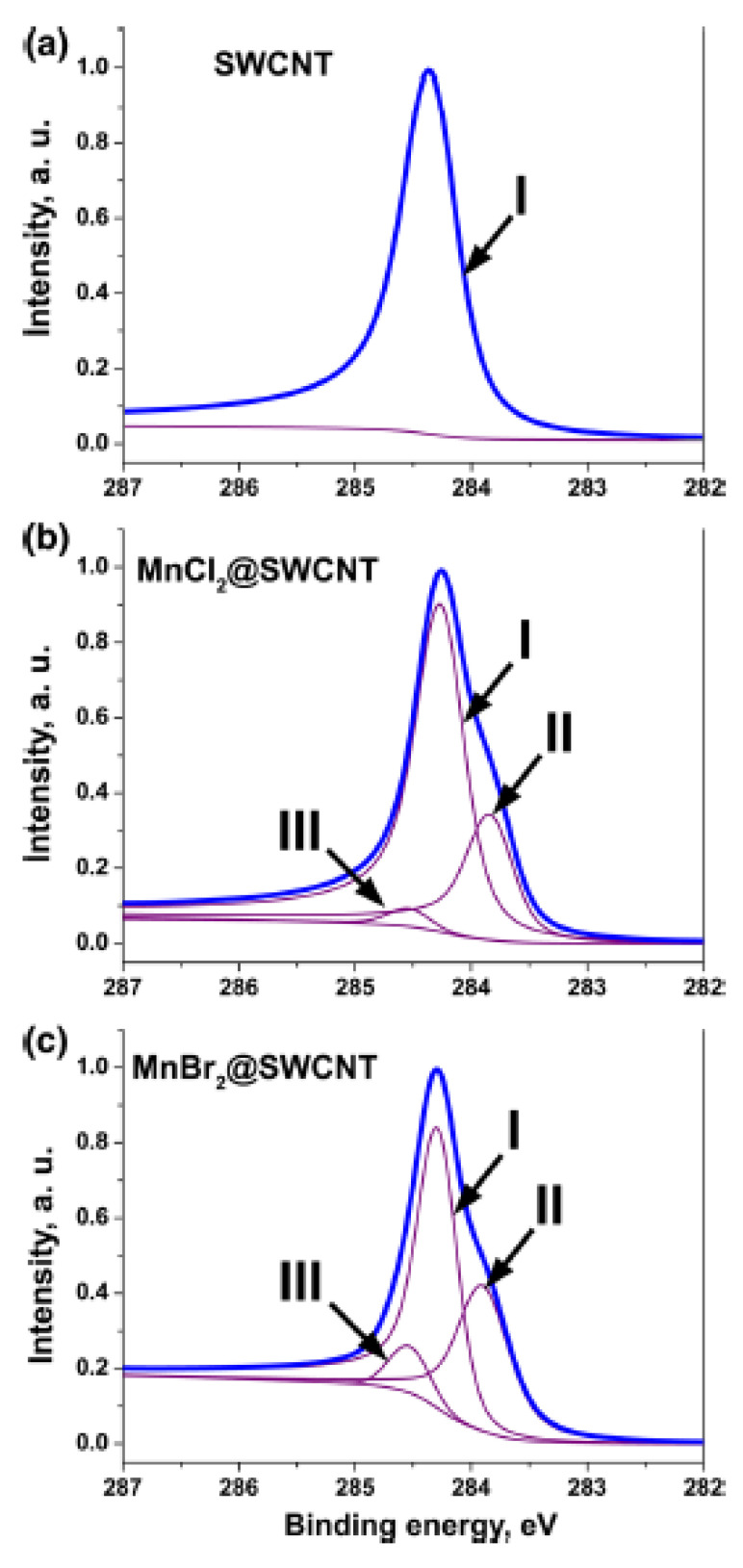
The C 1s XPS spectra of the pristine, and MnCl_2_, MnBr_2_ filled SWCNTs. The inset shows the shift in the peak. Reprinted from Kharlamova M.V. Electronic properties of single-walled carbon nanotubes filled with manganese halogenides. Appl. Phys. A. 2016. V.122. N.9. article number 791, Springer Nature [175].

XPS was employed to evaluate the direction and value of the Fermi level shift of the filled SWCNTs in Refs. [155,156,164,167,169,170,172,173,175,177,182,190,200,207]. The C 1s XPS spectra showed the shift in the peak and change in its width. The shift is caused by the charge transfer between SWCNTs and introduced compounds due to the Fermi level variation. The up- and downshift of SWCNTs corresponds to n- and p-doping of nanotubes by the encapsulated substances, respectively (Figure 8) [175]. UPS was used a direct method of investigation of the Fermi level shift in filled SWCNTs in Refs. [155,164,169]. The UPS spectra showed the shifts in the peaks of Van Hove singularities. This is evidence of the Fermi level shift in filled SWCNTs. The down- and upshift of the peaks corresponds to the lowering and increasing of the Fermi level of nanotubes. 

**Figure 9:**
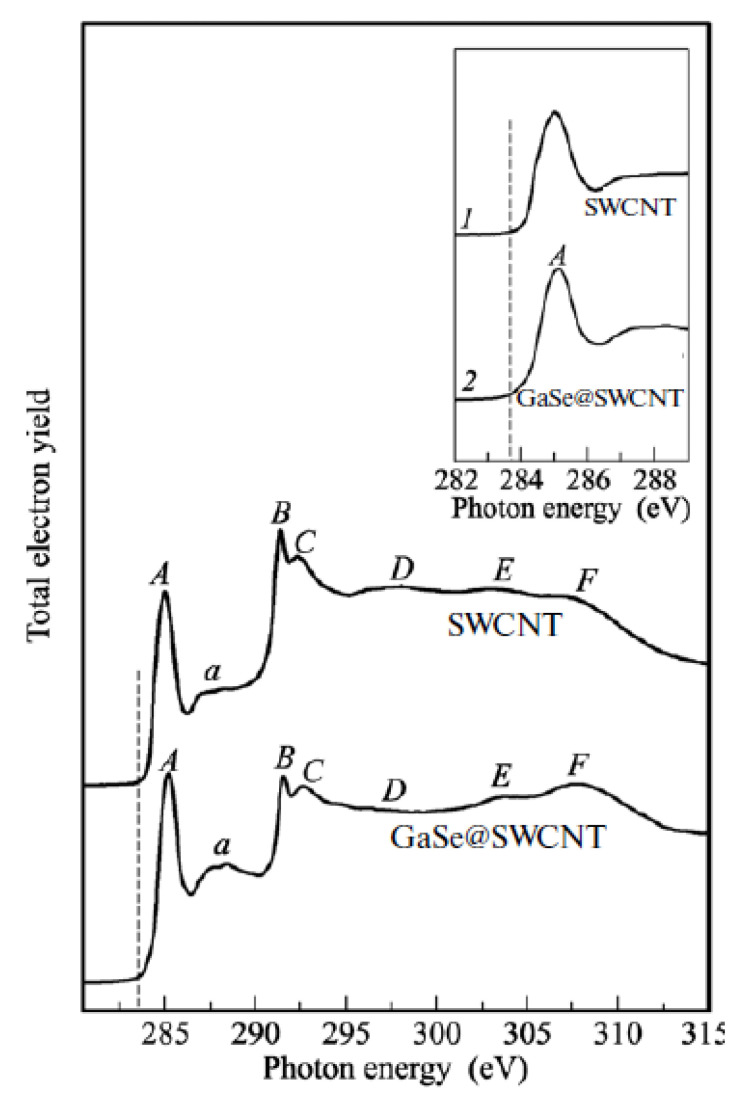
The NEXAFS spectra of the pristine and GaSe filled SWCNTs. Features of the spectra are denoted. The inset shows the π*-peak. Reprinted from Kharlamova M.V. Novel approach to tailoring the electronic properties of single-walled carbon nanotubes by the encapsulation of high-melting gallium selenide using a single-step process. JETP Letters. V.98. N.5. P.272–277, 2013, Springer Nature [212].

NEXAFS was applied to analyze local interactions between encapsulated substances and SWCNTs in Refs. [155,156,167,169,170,172,173,182,207]. The modifications in the spectra may testify to the formation of chemical bonds between the nanotube walls and introduced substances (Figure 9) [212]. 

Authors of Refs. [161,162] filled the separated metallic and semiconducting SWCNTs with copper chloride. Using optical absorption spectroscopy and Raman spectroscopy, they showed that the encapsulated salt leads to p-doping of nanotubes. The efficiency of doping for the metallic-enriched SWCNT fraction was higher than that for the semiconducting SWCNTs. The functionalization of both semiconducting and metallic SWCNT films by filling nanotubes with CuCl led to a significant increase in optical transmittance (above 90% in the near-infrared field). The best absolute optical transmittance was observed for filled metallic SWCNT films.

**Figure 10:**
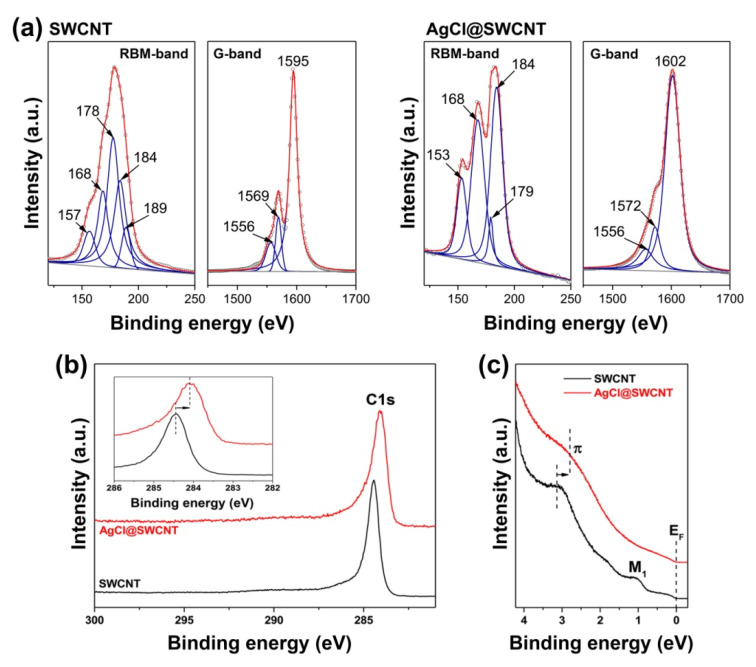
(**a**) The RBM and G-bands of Raman spectra of the pristine and silver (I) chloride-filled semiconducting SWCNTs acquired at a laser wavelength of 514 nm fitted with individual components. The RBM-band is fitted with the components belonging to the nanotubes of different diameters. The G-band is fitted with G^−^_LO_, G^+^_TO_ and G^+^_LO_ components [163]. Copyright 2018 M. V. Kharlamova et al. This is an open access article distributed under the Creative Commons Attribution License, which permits unrestricted use, distribution, and reproduction in any medium, provided the original work is properly cited. (**b**) The C 1s XPS spectra of the pristine and silver (I) chloride-filled metallic SWCNTs. The inset zooms in the shift in the C 1s peak, as shown by the arrow. The dashed vertical lines denote the peak positions. Reproduced from Kharlamova M.V. et al. Fermi level engineering of metallicity-sorted metallic single-walled carbon nanotubes by encapsulation of few-atom-thick crystals of silver (I) chloride. Journal of Materials Science. V.53. N.18. P.13,018–13,029. 2018, Springer Nature [164]. (**c**) The valence band spectra of the pristine and silver (I) chloride-filled metallic SWCNTs. The dashed vertical lines denote the positions of the π-peak. The shift in the π-peak is indicated by the arrow. The peak labeled M_1_ corresponds to the first Van Hove singularity of the pristine metallic SWCNTs. The Fermi level (E_F_) is indicated. Reproduced from Kharlamova M.V. et al. Fermi level engineering of metallicity-sorted metallic single-walled carbon nanotubes by encapsulation of few-atom-thick crystals of silver (I) chloride. Journal of Materials Science. V.53. N.18. P.13,018–13,029. 2018, Springer Nature [164].

In Ref. [163], high-purity semiconducting SWCNTs were filled with silver (I) chloride, and the multifrequency Raman spectra of filled and unfilled semiconducting SWCNTs were compared. Raman spectroscopy at five laser wavelengths allowed the investigating in detail of the electronic properties of the filled SWCNTs. Using different laser wavelengths allowed exciting electronic transitions of different semiconducting nanotubes and the study of the influence of the incorporated silver (I) chloride on their electronic properties. The RBM and G-bands of the Raman spectra of the pristine and filled SWCNTs were fitted with individual components. The peak positions and relative intensities of individual components of the RBM and G-bands of the pristine and filled SWCNTs were compared. Changes in peak intensities and appearance of new peaks in the RBM-band of the filled SWCNTs as compared to the pristine SWCNTs were revealed (Figure 10a). This testified to the filling-induced alteration in the resonance excitation conditions of nanotubes due to a charge transfer in the filled nanotubes that causes exciting optical transitions in smaller or larger diameter nanotubes using the laser with the same wavelength. Additionally, the prominent shifts in the components of the G-band of the filled SWCNTs as compared to the pristine SWCNTs as well as no changes in the component intensities were revealed (Figure 10a). The shifts in the components of the G-band were found to be independent of laser wavelength. These shifts were attributed to p-doping of SWCNTs by the inserted silver (I) chloride accompanied by the charge transfer from nanotubes to the salt.

Authors of Ref. [164] filled high-purity metallic SWCNTs with silver (I) chloride. The filled and unfilled SWCNTs were investigated by Raman spectroscopy, XPS and UPS, which were combined to study comprehensively the modified electronic properties of the filled SWCNTs. XPS confirmed the chemical composition of the encapsulated compound. A detailed analysis of Raman spectra of the pristine and filled SWCNTs allowed the investigation of filling-induced changes in the electronic properties of SWCNTs. The RBM and G-bands of the Raman spectra were fitted with individual components. The analysis of the RBM-band testified to the disappearance of the peak in the largest-diameter filled metallic SWCNTs, which was attributed to the changes in their resonance conditions. The analysis of the G-band revealed upshifts in the components of the filled SWCNTs and the changed profile of the band. This indicated doping of SWCNTs by the encapsulated silver (I) chloride and gap opening in the band structure of the filled SWCNTs, resulting in a transition of metallic nanotubes into semiconducting SWCNTs. The direction of the charge transfer in the filled SWCNTs was determined by XPS. The observed downshift of the C 1s XPS peak of the filled SWCNTs of 0.36 eV as well as its broadening were attributed to p-doping of SWCNTs accompanied by the charge transfer from the nanotubes to the inserted silver (I) chloride (Figure 10b). The detailed information on the doping-induced shift of the Fermi level of SWCNTs upon their filling was obtained by UPS. The UPS spectra of the filled SWCNTs demonstrated the downshift of the π-resonance by 0.36 eV, which was a direct confirmation of the downshift of the Fermi level of nanotubes (Figure 10c). 

The above-mentioned reports demonstrate the effect of the encapsulated materials on the electronic properties on either purely metallic or semiconducting SWCNTs. They also showcase the potential of precise Fermi level engineering of SWCNTs by filling their channels and achieving high doping levels. This control over the electronic properties will be the key to design the next generation of SWCNT-based nanoelectronic devices. 

Different encapsulated inorganic compounds have a different influence on the electronic properties of host SWCNTs. Several authors investigated the correlation between the chemical nature of the incorporated inorganic compound and their doping effect on nanotubes. 

**Figure 11:**
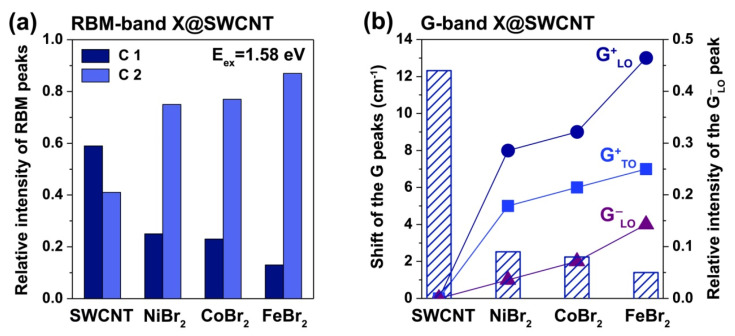
The results of the analysis of Raman spectra of the pristine SWCNTs and nanotubes filled with iron (II) bromide, cobalt (II) bromide and nickel (II) bromide. The comparison of the relative intensity in the RBM peaks (**a**), shifts of the G-band peaks and relative intensity of the G^−^_LO_ peak (**b**) [191]. Copyright 2015 Marianna V. Kharlamova. This is an open access article distributed under the Creative Commons Attribution License, which permits unrestricted use, distribution, and reproduction in any medium, provided the original work is properly cited.

In Ref. [191], a systematic comparison of the doping effect of encapsulated nickel (II) bromide, cobalt (II) bromide and iron (II) bromide on SWCNTs was conducted using Raman spectroscopy. The authors compared the relative intensities of and shifts in the peaks of the RBM and G-bands of the Raman spectra of the filled nanotubes (Figure 11). The RBM-band was fitted with two components corresponding to nanotubes of different diameters. In the spectrum of the pristine SWCNTs, the first component corresponding to the larger-diameter SWCNTs had a larger intensity. The relative intensity of two RBM peaks was changed upon the filling of nanotubes. In the spectra of the filled SWCNTs, the second component corresponding to the smaller-diameter SWCNTs had the larger intensity. The largest changes as compared to the pristine SWCNTs were observed for the SWCNTs filled with iron (II) bromide (Figure 11a). On the basis of these data, it was concluded that iron (II) bromide has a larger doping effect on SWCNTs than either cobalt (II) bromide or nickel (II) bromide. 

The results of the line shape analysis of the G-band of the Raman spectra of the filled SWCNTs were in agreement with the results of the fitting of the RBM-band. Figure 11b compares the shifts in the components of the G-bands and the relative intensities of the Breit−Wigner−Fano component (G^−^_LO_) for the pristine SWCNTs and filled nanotubes. It can be seen that the upshifts of all components of the G-band increase in line with nickel (II) bromide-cobalt (II) bromide-iron (II) bromide. In addition, the relative intensity of the metallic G^−^_LO_ component decreases significantly in the spectra of the filled SWCNTs as compared to the pristine nanotubes. The largest difference is again observed for the SWCNTs filled with iron (II) bromide. Taking into consideration these data, authors concluded that the iron (II) bromide has the largest p-doping effect on SWCNTs, whereas nickel (II) bromide has the smallest effect. This is explained by the different ionization potentials of iron, cobalt, and nickel. It is the minimal for iron, and it is maximal for nickel. Here the anion is the same, but the size of the anion and electron affinity of halogen plays an important role. 

In Ref. [183], a systematic comparison of the doping effect of the encapsulated terbium (III) chloride, terbium (III) bromide and terbium (III) iodide on SWCNTs was conducted using Raman spectroscopy. The fitting of the RBM and G-bands of the Raman spectra of the filled SWCNTs and comparison of the relative intensities of and shifts in the peaks showed that the p-doping effect on SWCNTs increases in line with terbium (III) iodide–terbium (III) bromide–terbium (III) chloride.

Combining the OAS, Raman spectroscopy and photoemission data allowed the authors of Refs. [155,156,177] to investigate in detail the influence of the anion type of the encapsulated metal halogenides on the doping level of SWCNTs. For halogenides of silver and copper, it was revealed that the p-doping level of SWCNTs increases in line with metal iodide–metal bromide–metal chloride [155,156]. For halogenides of lead, the strongest p-doping effect on SWCNTs was observed for lead (II) iodide, whereas the weakest p-doping effect was revealed for lead (II) bromide [177]. 

In Ref. [208], a comparison of the doping effect of the encapsulated tin, gallium and bismuth chalcogenides was performed using the data of OAS, Raman spectroscopy and XPS. It was shown that the encapsulation of tin sulfide inside the SWCNTs does not cause significant changes in the electronic structure of semiconducting SWCNTs, whereas there is a slight influence on metallic nanotubes. The introduction of gallium telluride inside the SWCNTs results in a downshift in the Fermi level of SWCNTs, i.e., p-doping of the nanotubes. The filling of SWCNTs with bismuth selenide does not lead to the modification of the electronic properties of nanotubes. 

## 5. Comparison of Doping Effect of Inorganic Compounds on Different Diameter SWCNTs

In Ref. [165], the authors performed a comparison of the doping levels of silver (I) chloride-filled metallicity-mixed SWCNTs with diameters of 1.4 and 1.9 nm synthesized by the arc-discharge and chemical vapor deposition methods, respectively. The electronic properties of the filled SWCNTs were investigated by Raman spectroscopy and XPS. The analysis of the Raman spectroscopy data acquired at laser wavelengths of 514 and 633 nm revealed the shifts and changes in the relative intensities of the components of the RBM-bands of the filled 1.4 nm-diameter SWCNTs, which are a result of the alteration in resonance excitation conditions of nanotubes upon their filling [165]. The RBM-band of the Raman spectrum of the filled 1.9 nm-diameter SWCNTs was completely suppressed; this was probably caused by the presence of large-diameter silver (I) chloride nanocrystal inside the SWCNT channel that distorted the radial symmetry of nanotubes. The G-band of the Raman spectra of the filled 1.4 and 1.9 nm-diameter SWCNTs showed significant upshifts, which were attributed to p-doping of SWCNTs by the encapsulated silver (I) chloride. In addition, the data indicated different filling ratios of 1.4 and 1.9 nm-diameter SWCNTs [165]. The XPS data confirmed that the filling of SWCNTs with silver (I) chloride led to the p-doping of nanotubes and downshift of their Fermi level. The C 1s XPS peaks of the filled 1.4 and 1.9 nm-diameter SWCNTs showed downshift values of 0.40 and 0.23 eV as compared to the pristine nanotubes, which may testify to the different doping levels of SWCNTs. Larger doping levels of the arc-discharge 1.4 nm-diameter SWCNTs as compared to the CVD 1.9 nm-diameter nanotubes were explained by the different filling ratios of SWCNTs, which are probably caused by differences in synthetic protocols of the pristine SWCNTs [165]. 

## 6. Discussion of the Influence of Different Encapsulated Substances on the Electronic Properties of SWCNTs

**Figure 12:**
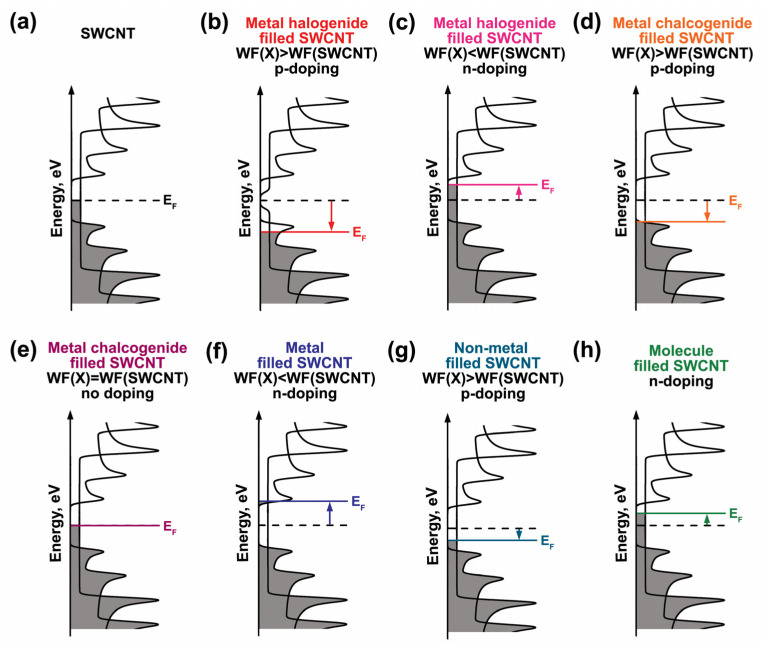
The schematics of the Fermi level shift in SWCNTs (**a**) filled with inorganic compounds–metal halogenides, whose work function (WF) is larger (**b**) or smaller (**c**) than the value of SWCNTs, and metal chalcogenides, whose work function is larger than (**d**) or equal (**e**) to the value of SWCNTs; elementary substances–metals (**f**) and non-metals (**g**), whose work functions are smaller or larger than the values of SWCNTs, respectively; and molecules (**h**).

The analysis of the literature shows that metal halogenides inside SWCNTs (Figure 12a), whose work function (WF) is larger (Figure 12b) or smaller (Figure 12c) than the value of SWCNTs lead to the downshift or upshift in the Fermi level of SWCNTs, accordingly. These metal halogenides are MHal (M=Ag, Cu, Hal=Cl, Br, I), MHal_2_ (M=Fe, Co, Ni, Mn, Zn, Cd, Pb, Hg, Hal = Cl, Br, I), MHal_3_ (M=Pr, Tb, Tm, Lu, Hal=Cl, Br, I) [154,155,156,157,158,159,160,161,162,163,164,165,166,167,168,169,170,171,172,173,174,175,176,177,178,179,180,181,182,183,184,185,186,187,188,189,190,191,192,193,194,195,196,197,198,199,200,201,202,203,204,205,206,207]. Metal chalcogenides, whose work function is larger (Figure 12d) or equal (Figure 12e) to the value of SWCNTs lead to the downshift in the Fermi level of SWCNTs, or no changes in the electronic structure of nanotubes, accordingly. These metal chalcogenides are MX (M=Ga, Sn, X=S, Se, Te), M_2×3_ (M=Bi, X=Se, Te) [208,209,210,211,212,213,214]. Elementary substances–metals (Figure 12f) and non-metals (Figure 12g), whose work functions are smaller or larger than the values of SWCNTs, or molecules (Figure 12h) result in the upshift or downshift of the Fermi level of SWCNTs, accordingly. These substances are Ag, Cu, Eu, P, As, I [45,46,47,48,49,50,51,52,53,54,55,56,57,58,59,60,61,62,63,64,65,66,67,68,69,70,71,72,73,74,75,76,77,78,79,80,81,82,83,84,85,86,87,88,89,90,91,92,93,94,95,96,97,98,99,100,101,102,103,104,105,106,107,108,109,110,111,112,113,114,115,116,117,118,119,120,121,122,123,124,125,126,127,128,129,130,131,132,133,134,135,136,137,138,139,140,141,142,143,144,145,146,147,148,149,150,151,152,153]. The discussion is as follows:

- Regarding kinetics of growth of carbon nanotubes, the growth mechanism of SWCNTs was revealed. The application of Raman spectroscopy allowed us to calculate the growth rates and activation energies. They are in the range of 0.5 to 3.0 eV. No activation energies for growth of individual carbon nanotubes were reported thus far.
- The authors showed the metal-dependence of growth kinetics, and that the use of different metal catalyst precursors allowed analyzing the influence of metal on the growth mechanism of SWCNTs. It was shown that different metals lead to different growth mechanisms of carbon nanotubes. More TEM investigations are required to analyze the mechanism in detail.
- Regarding the filling of metallicity-sorted SWCNTs, separated metallic, semiconducting SWCNT samples were proved to be a viable tool to unravel the effects of the filler on the electronic properties of the compound material. The investigation of the macroscopic electronic properties by a variety of spectroscopic methods (OAS, RS, XPS, UPS and XAS) allowed us to thoroughly address the influences on the electronic properties that are caused by encapsulated elementary substances, inorganic compounds and molecules.
- The authors developed new approaches to processing the data of spectroscopic investigations of filled SWCNTs. There have been developments in the evaluation of spectroscopic data. The precise and reliable data analysis supported drawing clear conclusions on the quantitative charge transfer present in differently filled SWCNTs.
- Regarding the correlation between the physical and chemical properties of encapsulated substances and the electronic properties of SWCNTs, the combined spectroscopic studies on filled SWCNTs allowed us to determine the charge transfer quantitatively. It can be evaluated as elementary charges per SWCNT surface atom, or additionally as elementary charges per SWCNT length. With the filling ratio and the interconversion of encapsulated substances, the doping level can be varied in a wide range. It is even possible to tune the doping level across the charge neutrality point and switch from n to p type doping. This powerful control of the electronic properties in heterogeneously filled SWCNTs enabled the demonstration of many different applications. The applications covered in this review include nanoelectronics, thermoelectric power generation, electrochemical energy storage, catalysis, sensors, spintronics, magnetic recording and biomedicine. This review also addresses current issues and likely contenders for a breakthrough in the near future, namely photovoltaics and light emission.

## 7. Conclusions

In this paper, we reviewed the kinetics of growth and the electronic properties of single-walled carbon nanotubes for applications. The use of methods of investigations allowed us to analyze in detail the properties of filled SWCNTs. New techniques of data processing were developed that allowed us to conclude about growth mechanisms and charge transfer mechanisms for filled SWCNTs toward applications. New samples were obtained, and their analysis confirmed the reported trends. Further progress is expected in controlled electronic properties of filled SWCNTs, which will, in conjunction with the wide range of demonstrated and envisaged applications, stimulate further advancements toward viable technologies built on them.

## Figures and Tables

**Table 1:** The encapsulated inorganic compounds, diameter and conductivity types of the host SWCNTs, methods of the investigation of the electronic properties, types of doping and the observed Fermi level shifts of SWCNTs.

Encapsulated Metal Halogenide	Diameter and Conductivity Type of SWCNTs ^a^	Methods of the Investigation of the Electronic Properties ^b^	Type of Doping	Fermi Level Shift, eV	Reference
AgCl	1.4 nm m-SWCNT	RS, XPS, UPS	p	−0.36	[164]
AgCl	1.4 nm s-SWCNT	RS	p	n/a	[163]
PrCl_3_	1.4 nm m-SWCNT	OAS, RS, XAS, XPS	p	−0.42	[182]
	1.4 nm s-SWCNT			−0.28	
CuCl	1.4 nm m-SWCNT	OAS, RS (+ EC charging), XAS, XPS, WF, VB	p	−0.57	[156]
	1.4 nm s-SWCNT			−0.37	
CuBr	1.4 nm m-SWCNT	OAS, RS (+ EC charging), XAS, XPS, WF, VB	p	−0.60	[156]
	1.4 nm s-SWCNT			−0.36	
CuI	1.4 nm m-SWCNT	OAS, RS (+ EC charging), XAS, XPS, WF, VB	p	−0.35	[156]
	1.4 nm s-SWCNT			−0.25	
MnCl_2_	1.4 nm mix-SWCNT	RS, XPS	p	−0.43	[175]
MnBr_2_	1.4 nm mix-SWCNT	RS, XPS	p	−0.37	[175]
FeCl_2_	1.4 nm mix-SWCNT	OAS, RS, XAS, XPS	p	−0.30	[170]
FeBr_2_	1.4 nm mix-SWCNT	OAS, RS, XAS, XPS	p	−0.30	[170]
FeI_2_	1.4 nm mix-SWCNT	OAS, RS, XAS, XPS	p	−0.30	[170]
CoBr_2_	1.4 nm mix-SWCNT	OAS, RS, XPS	p	−0.38	[190]
NiCl_2_	1.4 nm mix-SWCNT	OAS, RS, XAS, XPS	p	−0.58	[167]
NiBr_2_	1.4 nm mix-SWCNT	OAS, RS, XAS, XPS	p	−0.29	[167]
ZnCl_2_	1.4 nm mix-SWCNT	OAS, RS, XAS	p	n/a	[169]
ZnBr_2_	1.4 nm mix-SWCNT	OAS, RS, XAS, XPS, WF, VB	p	−0.28	[169]
ZnI_2_	1.4 nm mix-SWCNT	OAS, RS, XAS, XPS	p	−0.25	[169]
RbI	1.4 nm mix-SWCNT	RS, XPS	n	+0.20	[200]
RbAg_4_I_5_	1.4 nm mix-SWCNT	OAS, RS, XAS, XPS	p	−0.35	[207]
AgCl	1.4 nm mix-SWCNT	OAS, RS (+ EC charging), XAS, XPS, UPS	p	−0.38	[155]
AgBr	1.4 nm mix-SWCNT	OAS, RS (+ EC charging), XAS, XPS, UPS	p	−0.37	[155]
AgI	1.4 nm mix-SWCNT	OAS, RS (+ EC charging), XAS, XPS, UPS	p	−0.30	[155]
CdCl_2_	1.4 nm mix-SWCNT	OAS, RS, XAS, XPS	p	−0.36	[173]
CdBr_2_	1.4 nm mix-SWCNT	OAS, RS, XAS, XPS	p	−0.36	[173]
CdI_2_	1.4 nm mix-SWCNT	OAS, RS, XAS, XPS	p	−0.39	[173]
PbCl_2_	1.4 nm mix-SWCNT	RS, XPS	p	−0.15	[177]
PbBr_2_	1.4 nm mix-SWCNT	RS, XPS	p	−0.07	[177]
PbI_2_	1.4 nm mix-SWCNTs	RS, XPS	p	−0.17	[177]
TbCl_3_	1.4 nm mix-SWCNTs	RS	p		[183]
TbBr_3_	1.4 nm mix-SWCNTs	RS	p		[183]
TbI_3_	1.4 nm mix-SWCNTs	RS	p		[183]
TmCl_3_	1.4 nm mix-SWCNTs	RS	p		[211]
LuCl_3_	1.4 nm mix-SWCNTs	RS (+ EC charging)	p		[184]
LuBr_3_	1.4 nm mix-SWCNTs	RS (+ EC charging)	p		[184]
LuI_3_	1.4 nm mix-SWCNTs	RS (+ EC charging)	p		[184]
HgCl_2_	1.7 nm mix-SWCNT	XAS, XPS	p	−0.20	[172]

^a^ m-SWCNT = metallic SWCNTs, s-SWCNT = semiconducting SWCNTs, mix-SWCNT = metallicity-mixed SWCNTs; ^b^ OAS = optical absorption spectroscopy, RS (+ EC charging) = Raman spectroscopy (with electrochemical charging), XAS = X-ray absorption spectroscopy, XPS = X-ray photoelectron spectroscopy, UPS = ultraviolet photoelectron spectroscopy, WF = work function measurements, VB = valence band photoemission measurements.

## Data Availability

Data are available on request to the first author (M.V.K.) of every reviewed paper.

## References

[1] Kharlamova M.V. (2013). Electronic properties of pristine and modified single-walled carbon nanotubes. Phys.-Uspekhi..

[2] Ferguson V., Silva S.R.P., Zhang W. (2019). Carbon Materials in Perovskite Solar Cells: Prospects and Future Challenges. Energy Environ. Mater..

[3] Fukumaru T., Fujigaya T., Nakashima N. (2015). Development of n-type cobaltocene-encapsulated carbon nanotubes with remarkable thermoelectric property. Sci. Rep..

[4] Lee J.W., Jeon I., Lin H.S., Seo S., Han T.H., Anisimov A., Kauppinen E.I., Matsuo Y., Maruyama S., Yang Y. (2019). Vapor-Assisted Ex-Situ Doping of Carbon Nanotube toward Efficient and Stable Perovskite Solar Cells. Nano Lett..

[5] Jordan J.W., Lowe G.A., McSweeney R.L., Stoppiello C.T., Lodge R.W., Skowron S.T., Biskupek J., Rance G.A., Kaiser U., Walsh D.A. (2019). Host-Guest Hybrid Redox Materials Self-Assembled from Polyoxometalates and Single-Walled Carbon Nanotubes. Adv. Mater..

[6] Martincic M., Tobias G. (2014). Filled carbon nanotubes in biomedical imaging and drug delivery. Expert Opin. Drug Deliv..

[7] Liu B.L., Wu F.Q., Gui H., Zheng M., Zhou C.W. (2017). Chirality-Controlled Synthesis and Applications of Single-Wall Carbon Nanotubes. ACS Nano.

[8] Bati A.S.R., Yu L.P., Batmunkh M., Shapter J.G. (2019). Recent Advances in Applications of Sorted Single-Walled Carbon Nanotubes. Adv. Funct. Mater..

[9] Moore K.E., Tune D.D., Flavel B.S. (2015). Double-Walled Carbon Nanotube Processing. Adv. Mater..

[10] Kharlamova M.V. (2016). Advances in tailoring the electronic properties of single-walled carbon nanotubes. Prog. Mater. Sci..

[11] Kharlamova M.V., Kramberger C., Sato Y., Saito T., Suenaga K., Pichler T., Shiozawa H. (2018). Chiral vector and metal catalyst-dependent growth kinetics of single-wall carbon nanotubes. Carbon.

[12] Kharlamova M.V., Kramberger C., Saito T., Sato Y., Suenaga K., Picher T., Shiozawa H. (2017). Chirality-dependent growth of single-wall carbon nanotubes as revealed inside nano-test tubes. Nanoscale.

[13] Tkachenko N.V. (2006). Optical Spectroscopy: Methods and Instrumentations.

[14] Kharlamova M.V., Kramberger C., Sauer M., Yanagi K., Pichler T. (2015). Comprehensive spectroscopic characterization of high purity metallicity-sorted single-walled carbon nanotubes. Phys. Status Solidi (b).

[15] Ayala P., Miyata Y., De Blauwe K., Shiozawa H., Feng Y., Yanagi K., Kramberger C., Silva S.R.P., Follath R., Kataura H. (2009). Disentanglement of the electronic properties of metallicity-selected single-walled carbon nanotubes. Phys. Rev. B.

[16] Kuzmany H. (2009). Solid-State Spectroscopy: An Introduction.

[17] Atalla R.H., Agarwal U.P., Bond J.L. (1992). Raman spectroscopy. Springer series in wood science. Methods in Lignin Chemistry.

[18] Smekal A. (1923). The quantum theory of dispersion. Naturwissenschaften.

[19] Raman C.V., Krishnan K.S. (1928). The optical analog of the Compton eect. Nature.

[20] Lamdsberg G., Mandelstam L. (1928). A novel eect of light scattering in crystals. Naturwissenschaften.

[21] Maiman T.H. (1960). Stimulated optical radiation in ruby. Nature.

[22] Porto S.P.S., Wood D.L. (1962). Ruby optical maser as a raman source. J. Opt. Soc. Am..

[23] Smith E., Dent G. (2005). Modern Raman Spectroscopy: A Practical Approach.

[24] Dresselhaus M.S., Dresselhaus G., Jorio A., Souza A.G., Saito R. (2002). Raman spectroscopy on isolated single wall carbon nanotubes. Carbon.

[25] Araujo P.T., Maciel I.O., Pesce P.B.C., Pimenta M.A., Doorn S.K., Qian H., Hartschuh A., Steiner M., Grigorian L., Hata K. (2008). Nature of the constant factor in the relation between radial breathing mode frequency and tube diameter for single-wall carbon nanotubes. Phys. Rev. B..

[26] Fouquet M., Telg H., Maultzsch J., Wu Y., Chandra B., Hone J., Heinz T.F., Thomsen C. (2009). Longitudinal Optical Phonons in Metallic and Semiconducting Carbon Nanotubes. Phys. Rev. Lett..

[27] Brown S.D.M., Corio P., Marucci A., Dresselhaus M.S., Pimenta M.A., Kneipp K. (2000). Anti-Stokes Raman spectra of single-walled carbon nanotubes. Phys. Rev. B..

[28] Das A., Sood A.K. (2009). Renormalization of the phonon spectrum in semiconducting single-walled carbon nanotubes studied by Raman spectroscopy. Phys. Rev. B..

[29] Grimm S., Schiessl S.P., Zakharko Y., Rother M., Brohmann M., Zaumseil J. (2017). Doping-dependent G-mode shifts of small diameter semiconducting single-walled carbon nanotubes. Carbon.

[30] Tsang J.C., Freitag M., Perebeinos V., Liu J., Avouris P. (2007). Doping and phonon renormalization in carbon nanotubes. Nat. Nanotechnol..

[31] Das A., Sood A.K., Govindaraj A., Saitta A.M., Lazzeri M., Mauri F., Rao C.N.R. (2007). Doping in carbon nanotubes probed by Raman and transport measurements. Phys. Rev. Lett..

[32] Kalbac M., Farhat H., Kavan L., Kong J., Dresselhaus M.S. (2008). Competition between the Spring Force Constant and the Phonon Energy Renormalization in Electrochemically Doped Semiconducting Single-Walled Carbon Nanotubes. Nano Lett..

[33] Zhang L., Liao V., Yu Z.H. (2010). Raman spectroelectrochemistry of a single-wall carbon nanotube bundle. Carbon.

[34] Piscanec S., Lazzeri M., Robertson J., Ferrari A.C., Mauri F. (2007). Optical phonons in carbon nanotubes: Kohn anomalies, Peierls distortions, and dynamic effects. Phys. Rev. B..

[35] Lazzeri M., Piscanec S., Mauri F., Ferrari A.C., Robertson J. (2006). Phonon linewidths and electron-phonon coupling in graphite and nanotubes. Phys. Rev. B..

[36] Caudal N., Saitta A.M., Lazzeri M., Mauri F. (2007). Kohn anomalies and nonadiabaticity in doped carbon nanotubes. Phys. Rev. B..

[37] Nguyen K.T., Gaur A., Shim M. (2007). Fano lineshape and phonon softening in single isolated metallic carbon nanotubes. Phys. Rev. Lett..

[38] Farhat H., Son H., Samsonidze G.G., Reich S., Dresselhaus M.S., Kong J. (2007). Phonon softening in individual metallic carbon nanotubes due to the Kohn anomaly. Phys. Rev. Lett..

[39] Watts J.F., Wolstenholme J. (2003). An Introduction to Surface Analysis by XPS and AES..

[40] Leckrey R., O’Connor D.J., Sexton B.A., Smart R.S.C. (1992). Ultraviolet Photoelectron Spectroscopy of Solids. Surface Analysis Methods in Materials Science.

[41] Hertz H. (1887). Uber einen einuss des ultravioletten lichtes auf die electrische entladung. Ann. Phys..

[42] Einstein A. (1905). Uber einen die erzeugung und verwandlung des lichtes betreenden heuristischen gesichtspunkt. Ann. Phys..

[43] Innes P.D. (1907). On the velocity of the cathode particles emitted by various metals under the inuence of Röntgen rays, and its bearing on the theory of atomic disintegration. Proc. Roy. Soc. London Ser. A.

[44] Briggs D., Grant G.T. (2003). Perspectives on XPS and AES. Surface Analysis by Auger and X-ray Photoelectron Spectroscopy.

[45] Smith B.W., Monthioux M., Luzzi D.E. (1998). Encapsulated C-60 in carbon nanotubes. Nature.

[46] Botos A., Khlobystov A.N., Botka B., Hackl R., Szekely E., Simandi B., Kamaras K. (2010). Investigation of fullerene encapsulation in carbon nanotubes using a complex approach based on vibrational spectroscopy. Phys. Status Solidi (b).

[47] Burteaux B., Claye A., Smith B.W., Monthioux M., Luzzi D.E., Fischer J.E. (1999). Abundance of encapsulated C-60 in single-wall carbon nanotubes. Chem. Phys. Lett..

[48] Chamberlain T.W., Popov A.M., Knizhnik A.A., Samoilov G.E., Khlobystov A.N. (2010). The Role of Molecular Clusters in the Filling of Carbon Nanotubes. ACS Nano.

[49] Hirahara K., Suenaga K., Bandow S., Kato H., Okazaki T., Shinohara H., Iijima S. (2000). One-dimensional metallofullerene crystal generated inside single-walled carbon nanotubes. Phys. Rev. Lett..

[50] Jeong G.H., Hirata T., Hatakeyama R., Tohji K., Motomiya K. (2002). C-60 encapsulation inside single-walled carbon nanotubes using alkali-fullerene plasma method. Carbon.

[51] Kataura H., Maniwa Y., Kodama T., Kikuchi K., Hirahara K., Suenaga K., Iijima S., Suzuki S., Achiba Y., Kratschmer W. (2001). High-yield fullerene encapsulation in single-wall carbon nanotubes. Synth. Met..

[52] Kataura H., Maniwa Y., Abe M., Fujiwara A., Kodama T., Kikuchi K., Imahori H., Misaki Y., Suzuki S., Achiba Y. (2002). Optical properties of fullerene and non-fullerene peapods. Appl. Phys. A.

[53] Khlobystov A.N., Britz D.A., Wang J.W., O’Neil S.A., Poliakoff M., Briggs G.A.D. (2004). Low temperature assembly of fullerene arrays in single-walled carbon nanotubes using supercritical fluids. J. Mater. Chem..

[54] Khlobystov A.N., Porfyrakis K., Kanai M., Britz D.A., Ardavan A., Shinohara H., Dennis T.J.S., Briggs G.A.D. (2004). Molecular motion of endohedral fullerenes in single-walled carbon nanotubes. Aew. Chem. Int. Ed..

[55] Khlobystov A.N., Britz D.A., Briggs G.A.D. (2005). Molecules in carbon nanotubes. Accounts Chem. Res..

[56] Luzzi D.E., Smith B.W. (2000). Carbon cage structures in single wall carbon nanotubes: A new class of materials. Carbon.

[57] Monthioux M., Smith B.W., Burteaux B., Claye A., Fischer J.E., Luzzi D.E. (2001). Sensitivity of single-wall carbon nanotubes to chemical processing: An electron microscopy investigation. Carbon.

[58] Shimada T., Ohno Y., Okazaki T., Sugai T., Suenaga K., Kishimoto S., Mizutani T., Inoue T., Taniguchi R., Fukui N. (2004). Transport properties of C-78, C-90 and Dy@C-82 fullerenes-nanopeapods by field effect transistors. Physica E.

[59] Shiozawa H., Ishii H., Kihara H., Sasaki N., Nakamura S., Yoshida T., Takayama Y., Miyahara T., Suzuki S., Achiba Y. (2006). Photoemission and inverse photoemission study of the electronic structure of C-60 fullerenes encapsulated in single-walled carbon nanotubes. Phys. Rev. B.

[60] Simon F., Kuzmany H., Rauf H., Pichler T., Bernardi J., Peterlik H., Korecz L., Fulop F., Janossy A. (2004). Low temperature fullerene encapsulation in single wall carbon nanotubes: Synthesis of N@C-60@SWCNT. Chem. Phys. Lett..

[61] Sloan J., Dunin-Borkowski R.E., Hutchison J.L., Coleman K.S., Williams V.C., Claridge J.B., York A.P.E., Xu C.G., Bailey S.R., Brown G. (2000). The size distribution, imaging and obstructing properties of C-60 and higher fullerenes formed within arc-grown single walled carbon nanotubes. Chem. Phys. Lett..

[62] Smith B.W., Monthioux M., Luzzi D.E. (1999). Carbon nanotube encapsulated fullerenes: A unique class of hybrid materials. Chem. Phys. Lett..

[63] Smith B.W., Luzzi D.E. (2000). Formation mechanism of fullerene peapods and coaxial tubes: A path to large scale synthesis. Chem. Phys. Lett..

[64] Yudasaka M., Ajima K., Suenaga K., Ichihashi T., Hashimoto A., Iijima S. (2003). Nano-extraction and nano-condensation for C-60 incorporation into single-wall carbon nanotubes in liquid phases. Chem. Phys. Lett..

[65] Zhang Y., Iijima S., Shi Z., Gu Z. (1999). Defects in arc-discharge-produced single-walled carbon nanotubes. Philos. Mag. Lett..

[66] Simon F., Kramberger C., Pfeiffer R., Kuzmany H., Zolyomi V., Kurti J., Singer P.M., Alloul H. (2005). Isotope engineering of carbon nanotube systems. Phys. Rev. Lett..

[67] Simon F., Kukovecz A., Kramberger C., Pfeiffer R., Hasi F., Kuzmany H., Kataura H. (2005). Diameter selective reaction processes of single-wall carbon nanotubes. Phys. Rev. B.

[68] Ashino M., Obergfell D., Haluska M., Yang S.H., Khlobystov A.N., Roth S., Wiesendanger R. (2008). Atomically resolved mechanical response of individual metallofullerene molecules confined inside carbon nanotubes. Nat. Nanotechnol..

[69] Chiu P.W., Gu G., Kim G.T., Philipp G., Roth S., Yang S.F., Yang S. (2001). Temperature-induced change from *p* to *n* conduction in metallofullerene nanotube peapods. Appl. Phys. Lett..

[70] Gloter A., Suenaga K., Kataura H., Fujii R., Kodama T., Nishikawa H., Ikemoto I., Kikuchi K., Suzuki S., Achiba Y. (2004). Structural evolutions of carbon nano-peapods under electron microscopic observation. Chem. Phys. Lett..

[71] Kitaura R., Imazu N., Kobayashi K., Shinohara H. (2008). Fabrication of metal nanowires in carbon nanotubes via versatile nano-template reaction. Nano Lett..

[72] Okazaki T., Suenaga K., Hirahara K., Bandow S., Iijima S., Shinohara I.E. (2001). Real time reaction dynamics in carbon nanotubes. J. Am. Chem. Soc..

[73] Okazaki T., Suenaga K., Hirahara K., Bandow S., Iijima S., Shinohara H. (2002). Electronic and geometric structures of metallofullerene peapods. Phys. B Condens. Matter.

[74] Pichler T., Kramberger C., Ayala P., Shiozawa H., Knupfer M., Rummeli M.H., Batchelor D., Kitaura R., Imazu N., Kobayashi K. (2008). Bonding environment and electronic structure of Gd metallofullerene and Gd nanowire filled single-wall carbon nanotubes. Phys. Status Solidi (b).

[75] Suenaga K., Tence T., Mory C., Colliex C., Kato H., Okazaki T., Shinohara H., Hirahara K., Bandow S., Iijima S. (2000). Element-selective single atom imaging. Science.

[76] Ayala P., Kitaura R., Kramberger C., Shiozawa H., Imazu N., Kobayashi K., Mowbray D.J., Hoffmann P., Shinohara H., Pichler T. (2011). A Resonant Photoemission Insight to the Electronic Structure of Gd Nanowires Templated in the Hollow Core of SWCNTs. Mater. Express.

[77] Debarre A., Jaffiol R., Julien C., Richard A., Nutarelli D., Tchenio P. (2003). Antenna effect in dimetallofullerene peapods. Chem. Phys. Lett..

[78] Smith B.W., Luzzi D.E., Achiba Y. (2000). Tumbling atoms and evidence for charge transfer in La-2@C-80@SWNT. Chem. Phys. Lett..

[79] Suenaga K., Okazaki T., Wang C.R., Bandow S., Shinohara H., Iijima S. (2003). Direct imaging of Sc-2@C-84 molecules encapsulated inside single-wall carbon nanotubes by high resolution electron microscopy with atomic sensitivity. Phys. Rev. Lett..

[80] Suenaga K., Taniguchi R., Shimada T., Okazaki T., Shinohara H., Iijima S. (2003). Evidence for the intramolecular motion of Gd atoms in a Gd-2@C-92 nanopeapod. Nano Lett..

[81] Britz D.A., Khlobystov A.N., Porfyrakis K., Ardavan A., Briggs G.A.D. (2004). Chemical reactions inside single-walled carbon nano test-tubes. Chem. Commun..

[82] Sun B.Y., Sato Y., Suenaga K., Okazaki T., Kishi N., Sugai T., Bandow S., Iijima S., Shinohara H. (2005). Entrapping of exohedral metallofullerenes in carbon nanotubes: (CsC60)(n)@SWNT nano-peapods. J. Am. Chem. Soc..

[83] Chamberlain T.W., Champness N.R., Schroder M., Khlobystov A.N. (2010). A Piggyback Ride for Transition Metals: Encapsulation of Exohedral Metallofullerenes in Carbon Nanotubes. Chem.–A Eur. J..

[84] Chamberlain T.W., Meyer J.C., Biskupek J., Leschner J., Santana A., Besley N.A., Bichoutskaia E., Kaiser U., Khlobystov A.N. (2011). Reactions of the inner surface of carbon nanotubes and nanoprotrusion processes imaged at the atomic scale. Nat. Chem..

[85] Khlobystov A.N. (2011). Carbon Nanotubes: From Nano Test Tube to Nano-Reactor. Acs Nano.

[86] Britz D.A., Khlobystov A.N., Wang J.W., O’Neil A.S., Poliakoff M., Ardavan A., Briggs G.A.D. (2004). Selective host-guest interaction of single-walled carbon nanotubes with functionalised fullerenes. Chem. Commun..

[87] Briones A., Liu X.J., Kramberger C., Saito T., Pichler T. (2011). Nanochemical reactions by laser annealing of ferrocene filled single-walled carbon nanotubes. Phys. Status Solidi (b).

[88] Guan L.H., Shi Z.J., Li M.X., Gu Z.N. (2005). Ferrocene-filled single-walled carbon nanotubes. Carbon.

[89] Kocsis D., Kaptas D., Botos A., Pekker A., Kamaras K. (2011). Ferrocene encapsulation in carbon nanotubes: Various methods of filling and investigation. Phys. Status Solidi (b).

[90] Liu X.J., Kuzmany H., Saito T., Pichler T. (2011). Temperature dependence of inner tube growth from ferrocene-filled single-walled carbon nanotubes. Phys. Status Solidi (b).

[91] Liu X.J., Kuzmany H., Ayala P., Calvaresi M., Zerbetto F., Pichler T. (2012). Selective Enhancement of Photoluminescence in Filled Single-Walled Carbon Nanotubes. Adv. Funct. Mater..

[92] Plank W., Kuzmany H., Pfeiffer R., Saito T., Iijima S. (2009). Raman scattering from ferrocene encapsulated in narrow diameter carbon nanotubes. Phys. Status Solidi (b).

[93] Sauer M., Shiozawa H., Ayala P., Ruiz-Soria G., Kataura H., Yanagi K., Krause S., Pichler T. (2012). In situ filling of metallic single-walled carbon nanotubes with ferrocene molecules. Phys. Status Solidi (b).

[94] Shiozawa H., Pichler T., Pfeiffer R., Kuzmany H., Kataura H. (2007). Ferrocene encapsulated in single-wall carbon nanotubes: A precursor to secondary tubes. Phys. Status Solidi (b).

[95] Shiozawa H., Pichler T., Kramberger C., Gruneis A., Knupfer M., Buchner B., Zolyomi V., Koltai J., Kurti J., Batchelor D. (2008). Fine tuning the charge transfer in carbon nanotubes via the interconversion of encapsulated molecules. Phys. Rev. B.

[96] Shiozawa H., Giusca C.E., Silva S.R.P., Kataura H., Pichler T. (2008). Capillary filling of single-walled carbon nanotubes with ferrocene in an organic solvent. Phys. Status Solidi (b).

[97] Shiozawa H., Pichler T., Gruneis A., Pfeiffer R., Kuzmany H., Liu Z., Suenaga K., Kataura H. (2008). A catalytic reaction inside a single-walled carbon nanotube. Adv. Mater..

[98] Shiozawa H., Kramberger C., Pfeiffer R., Kuzmany H., Pichler T., Liu Z., Suenaga K., Kataura H., Silva S.R.P. (2010). Catalyst and Chirality Dependent Growth of Carbon Nanotubes Determined Through Nano-Test Tube Chemistry. Adv. Mater..

[99] Kharlamova M.V., Sauer M., Saito T., Krause S., Liu X., Yanagi K., Pichler T., Shiozawa H. (2013). Inner tube growth properties and electronic structure of ferrocene-filled large diameter single-walled carbon nanotubes. Phys. Status Solidi (b).

[100] Kharlamova M.V., Kramberger C., Saito T., Shiozawa H., Pichler T. (2014). In situ Raman spectroscopy studies on time-dependent inner tube growth in ferrocene-filled large diameter single-walled carbon nanotubes. Phys. Status Solidi (b).

[101] Sauer M., Shiozawa H., Ayala P., Ruiz-Soria G., Liu X.J., Chernov A., Krause S., Yanagi K., Kataura H., Pichler T. (2013). Internal charge transfer in metallicity sorted ferrocene filled carbon nanotube hybrids. Carbon.

[102] Plank W., Pfeiffer R., Schaman C., Kuzmany H., Calvaresi M., Zerbetto F., Meyer J. (2010). Electronic Structure of Carbon Nanotubes with Ultrahigh Curvature. ACS Nano.

[103] Briones-Leon A., Ayala P., Liu X.J., Yanagi K., Weschke E., Eisterer M., Jiang H., Kataura H., Pichler T., Shiozawa H. (2013). Orbital and spin magnetic moments of transforming one-dimensional iron inside metallic and semiconducting carbon nanotubes. Phys. Rev. B.

[104] Li L.J., Khlobystov A.N., Wiltshire J.G., Briggs G.A.D., Nicholas R.J. (2005). Diameter-selective encapsulation of metallocenes in single-walled carbon nanotubes. Nat. Mater..

[105] Kharlamova M.V., Kramberger C., Saito T., Shiozawa H., Pichler T. (2016). Growth dynamics of inner tubes inside cobaltocene-filled single-walled carbon nanotubes. Appl. Phys. A..

[106] Kharlamova M.V., Sauer M., Saito T., Sato Y., Suenaga K., Pichler T., Shiozawa H. (2014). Doping of single-walled carbon nanotubes controlled via chemical transformation of encapsulated nickelocene. Nanoscale.

[107] Kharlamova M.V., Sauer M., Egorov A., Kramberger C., Saito T., Pichler T., Shiozawa H. (2015). Temperature-dependent inner tube growth and electronic structure of nickelocene-filled single-walled carbon nanotubes. Phys. Status Solidi (b).

[108] Kharlamova M.V., Kramberger C., Sauer M., Yanagi K., Saito T., Pichler T. (2018). Inner tube growth and electronic properties of metallicity-sorted nickelocene-filled semiconducting single-walled carbon nanotubes. Appl. Phys. A.

[109] Shiozawa H., Pichler T., Kramberger C., Rummeli M., Batchelor D., Liu Z., Suenaga K., Kataura H., Silva S.R.P. (2009). Screening the Missing Electron: Nanochemistry in Action. Phys. Rev. Lett..

[110] Shiozawa H., Kramberger C., Rummeli M., Batchelor D., Kataura H., Pichler T., Silva S.R.P. (2009). Electronic properties of single-walled carbon nanotubes encapsulating a cerium organometallic compound. Phys. Status Solidi (b).

[111] Shiozawa H., Silva S.R.P., Liu Z., Suenaga K., Kataura H., Kramberger C., Pfeiffer R., Kuzmany H., Pichler T. (2010). Low-temperature growth of single-wall carbon nanotubes inside nano test tubes. Phys. Status Solidi (b).

[112] Stoppiello C.T., Biskupek J., Li Z.Y., Rance G.A., Botos A., Fogarty R.M., Bourne R.A., Yuan J., Lovelock K.R.J., Thompson P. (2017). A one-pot-one-reactant synthesis of platinum compounds at the nanoscale. Nanoscale.

[113] Sauer M., Briones-Leon A., Saito T., Yanagi K., Schulte K., Pichler T., Shiozawa H. (2015). Tailoring the electronic properties of single-walled carbon nanotubes via filling with nickel acetylacetonate. Phys. Status Solidi (b).

[114] Loebick C.Z., Majewska M., Ren F., Haller G.L., Pfefferle L.D. (2010). Fabrication of Discrete Nanosized Cobalt Particles Encapsulated Inside Single-Walled Carbon Nanotubes. J. Phys. Chem. C.

[115] Morgan D.A., Sloan J., Green M.L.H. (2002). Direct imaging of o-carborane molecules within single walled carbon nanotubes. Chem. Commun..

[116] Kataura H., Maniwa Y., Kodama T., Kikuchi K., Suzuki S., Achiba Y., Sugiura K., Okubo S., Tsukagoshi K. (2003). One-dimensional system in carbon nanotubes. AIP Conf. Proc..

[117] Tschirner N., Brose K., Maultzsch J., Yanagi K., Kataura H., Thomsen C. (2010). The influence of incorporated beta-carotene on the vibrational properties of single wall carbon nanotubes. Phys. Status Solidi (b).

[118] Yanagi K., Miyata Y., Kataura H. (2006). Highly stabilized beta-carotene in carbon nanotubes. Adv. Mater..

[119] Takenobu T., Takano T., Shiraishi M., Murakami Y., Ata M., Kataura H., Achiba Y., Iwasa Y. (2003). Stable and controlled amphoteric doping by encapsulation of organic molecules inside carbon nanotubes. Nat. Mater..

[120] Fan X., Dickey E.C., Eklund P.C., Williams K.A., Grigorian L., Buczko R., Pantelides S.T., Pennycook S.J. (2000). Atomic arrangement of iodine atoms inside single-walled carbon nanotubes. Phys. Rev. Lett..

[121] Guan L.H., Suenaga K., Shi Z.J., Gu Z.N., Iijima S. (2007). Polymorphic structures of iodine and their phase transition in confined nanospace. Nano Lett..

[122] Kissell K.R., Hartman K.B., Van der Heide P.A.W., Wilson L.J. (2006). Preparation of I-2@ SWNTs: Synthesis and spectroscopic characterization of I-2-loaded SWNTs. J. Phys. Chem. B.

[123] Tonkikh A.A., Tsebro V.I., Obraztsova E.A., Suenaga K., Kataura H., Nasibulin A.G., Kauppinen E.I., Obraztsova E.D. (2015). Metallization of single-wall carbon nanotube thin films induced by gas phase iodination. Carbon.

[124] Hatakeyama R., Li Y. (2007). Synthesis and electronic-property control of Cs-encapsulated single- and double-walled carbon nanotubes by plasma ion irradiation. J. Appl. Phys..

[125] Jeong G.H., Hatakeyama R., Hirata T., Tohji K., Motomiya K., Yaguchi T., Kawazoe Y. (2003). Formation and structural observation of cesium encapsulated single-walled carbon nanotubes. Chem. Commun..

[126] Nishide D., Dohi H., Wakabayashi T., Nishibori E., Aoyagi S., Ishida M., Kikuchi S., Kitaura R., Sugai T., Sakata M. (2006). Single-wall carbon nanotubes encaging linear chain C10H2 polyyne molecules inside. Chem. Phys. Lett..

[127] Chancolon J., Archaimbault F., Pineau A., Bonnamy S. (2006). Filling of carbon nanotubes with selenium by vapor phase process. J. Nanosci. Nanotech..

[128] Chernysheva M.V., Kiseleva E.A., Verbitskii N.I., Eliseev A.A., Lukashin A.V., Tretyakov Y.D., Savilov S.V., Kiselev N.A., Zhigalina O.M., Kumskov A.S. (2008). The electronic properties of SWNTs intercalated by electron acceptors. Physica E.

[129] Sedelnikova O.V., Gurova O.A., Makarova A.A., Fedorenko A.D., Nikolenko A.D., Plyusnin P.E., Arenal R., Bulusheva L.G., Okotrub A.V. (2020). Light-Induced Sulfur Transport inside Single-Walled Carbon Nanotubes. Nanomaterials.

[130] Hart M., White E.R., Chen J., McGilvery C.M., Pickard C.J., Michaelides A., Sella A., Shaffer M.S.P., Salzmann C.G. (2017). Encapsulation and Polymerization of White Phosphorus Inside Single-Wall Carbon Nanotubes. Aew. Chem. Int. Edit..

[131] Hart M., Chen J., Michaelides A., Sella A., Shaffer M.S.P., Salzmann C.G. (2018). One-Dimensional Arsenic Allotropes: Polymerization of Yellow Arsenic Inside Single-Wall Carbon Nanotubes. Aew. Chem. Int. Edit..

[132] Wang Z.Y., Shi Z.J., Gu Z.N. (2010). Synthesis of single-walled carbon nanotube/metal nanoparticle hybrid materials from potassium-filled nanotubes. Carbon.

[133] Kiang C.H., Choi J.S., Tran T.T., Bacher A.D. (1999). Molecular nanowires of 1 nm diameter from capillary filling of single-walled carbon nanotubes. J. Phys. Chem. B.

[134] Borowiak-Palen E., Mendoza E., Bachmatiuk A., Rummeli M.H., Gemming T., Nogues J., Skumryev V., Kalenczuk R.J., Pichler T., Silva S.R.P. (2006). Iron filled single-wall carbon nanotubes-A novel ferromagnetic medium. Chem. Phys. Lett..

[135] Borowiak-Palen E., Bachmatiuk A., Rummeli M.H., Gemming T., Pichler T., Kalenczuk R.J. (2006). Iron filled singlewalled carbon nanotubes-synthesis and characteristic properties. Phys. Status Solidi (b).

[136] Cui T.T., Pan X.L., Dong J.H., Miao S., Miao D.Y., Bao X.H. (2018). A versatile method for the encapsulation of various non-precious metal nanoparticles inside single-walled carbon nanotubes. Nano Res..

[137] Li Y.F., Kaneko T., Ogawa T., Takahashi M., Hatakeyama R. (2008). Novel properties of single-walled carbon nanotubes with encapsulated magnetic atoms. Jpn. J. Appl. Phys..

[138] Domanov O., Weschke E., Saito T., Peterlik H., Pichler T., Eisterer M., Shiozawa H. (2019). Exchange coupling in a frustrated trimetric molecular magnet reversed by a 1D nano-confinement. Nanoscale.

[139] Sloan J., Hammer J., Zwiefka-Sibley M., Green M.L.H. (1998). The opening and filling of single walled carbon nanotubes (SWTs). Chem. Commun..

[140] Govindaraj A., Satishkumar B.C., Nath M., Rao C.N.R. (2000). Metal nanowires and intercalated metal layers in single-walled carbon nanotube bundles. Chem. Mater..

[141] Kharlamova M.V., Niu J.J. (2012). Comparison of metallic silver and copper doping effects on single-walled carbon nanotubes. Appl. Phys. A.

[142] Kharlamova M.V., Niu J.J. (2012). Donor doping of single-walled carbon nanotubes by filling of channels with silver. J. Exp. Theor. Phys..

[143] Borowiak-Palen E., Ruemmeli M.H., Gemming T., Pichler T., Kalenczuk R.J., Silva S.R.P. (2006). Silver filled single-wall carbon nanotubes-synthesis, structural and electronic properties. Nanotechnology.

[144] Corio P., Santos A.P., Santos P.S., Temperini M.L.A., Brar V.W., Pimenta M.A., Dresselhaus M.S. (2004). Characterization of single wall carbon nanotubes filled with silver and with chromium compounds. Chem. Phys. Lett..

[145] Sloan J., Wright D.M., Woo H.G., Bailey S., Brown G., York A.P.E., Coleman K.S., Hutchison J.L., Green M.L.H. (1999). Capillarity and silver nanowire formation observed in single walled carbon nanotubes. Chem. Commun..

[146] Zhang Z.L., Li B., Shi Z.J., Gu Z.N., Xue Z.Q., Peng L.M. (2000). Filling of single-walled carbon nanotubes with silver. J. Mater. Res..

[147] Kharlamova M.V., Niu J.J. (2012). New method of the directional modification of the electronic structure of single-walled carbon nanotubes by filling channels with metallic copper from a liquid phase. JETP Lett..

[148] Chamberlain T.W., Zoberbier T., Biskupek J., Botos A., Kaiser U., Khlobystov A.N. (2012). Formation of uncapped nanometre-sized metal particles by decomposition of metal carbonyls in carbon nanotubes. Chem. Sci..

[149] Costa P.M.F.J., Sloan J., Rutherford T., Green M.L.H. (2005). Encapsulation of RexOy clusters within single-walled carbon nanotubes and their in tubulo reduction and sintering to Re metal. Chem. Mater..

[150] Zoberbier T., Chamberlain T.W., Biskupek J., Kuganathan N., Eyhusen S., Bichoutskaia E., Kaiser U., Khlobystov A.N. (2012). Interactions and Reactions of Transition Metal Clusters with the Interior of Single-Walled Carbon Nanotubes Imaged at the Atomic Scale. J. Am. Chem. Soc..

[151] Kitaura R., Nakanishi R., Saito T., Yoshikawa H., Awaga K., Shinohara H. (2009). High-Yield Synthesis of Ultrathin Metal Nanowires in Carbon Nanotubes. Aew. Chem. Int. Edit..

[152] Nakanishi R., Kitaura R., Ayala P., Shiozawa H., De Blauwe K., Hoffmann P., Choi D., Miyata Y., Pichler T., Shinohara H. (2012). Electronic structure of Eu atomic wires encapsulated inside single-wall carbon nanotubes. Phys. Rev. B.

[153] Ayala P., Kitaura R., Nakanishi R., Shiozawa H., Ogawa D., Hoffmann P., Shinohara H., Pichler T. (2011). Templating rare-earth hybridization via ultrahigh vacuum annealing of ErCl3 nanowires inside carbon nanotubes. Phys. Rev. B.

[154] Zakalyukin R.M., Mavrin B.N., Dem’yanets L.N., Kiselev N.A. (2008). Synthesis and characterization of single-walled carbon nanotubes filled with the superionic material SnF2. Carbon.

[155] Eliseev A.A., Yashina L.V., Brzhezinskaya M.M., Chernysheva M.V., Kharlamova M.V., Verbitsky N.I., Lukashin A.V., Kiselev N.A., Kumskov A.S., Zakalyuhin R.M. (2010). Structure and electronic properties of AgX (X = Cl, Br, I)-intercalated single-walled carbon nanotubes. Carbon.

[156] Eliseev A.A., Yashina L.V., Verbitskiy N.I., Brzhezinskaya M.M., Kharlamova M.V., Chernysheva M.V., Lukashin A.V., Kiselev N.A., Kumskov A.S., Freitag B. (2012). Interaction between single walled carbon nanotube and 1D crystal in CuX@SWCNT (X = Cl, Br, I) nanostructures. Carbon.

[157] Flahaut E., Sloan J., Coleman K., Green M. (2001). Synthesis of 1D P-block halide crystals within single walled carbon nanotubes. AIP Conf. Proc..

[158] Monthioux M., Flahaut E., Cleuziou J.P. (2006). Hybrid carbon nanotubes: Strategy, progress, and perspectives. J. Mater. Res..

[159] Sloan J., Kirkland A.I., Hutchison J.L., Green M.L.H. (2002). Integral atomic layer architectures of 1D crystals inserted into single walled carbon nanotubes. Chem. Commun..

[160] Eremina V.A., Fedotov P.V., Obraztsova E.D. (2015). Copper chloride functionalization of semiconducting and metallic fractions of single-walled carbon nanotubes. J. Nanophotonics.

[161] Fedotov P.V., Tonkikh A.A., Obraztsova E.A., Nasibulin A.G., Kauppinen E.I., Chuvilin A.L., Obraztsova E.D. (2014). Optical properties of single-walled carbon nanotubes filled with CuCl by gas-phase technique. Phys. Status Solidi (b).

[162] Fedotov P.V., Eremina V.A., Tonkikh A.A., Chernov A.I., Obraztsova E.D. (2016). Enhanced optical transparency of films formed from sorted metallic or semiconducting single-walled carbon nanotubes filled with CuCl. Phys. Status Solidi (b).

[163] Kharlamova M.V., Kramberger C., Mittelberger A., Yanagi K., Pichler T., Eder D. (2018). Silver Chloride Encapsulation-Induced Modifications of Raman Modes of Metallicity-Sorted Semiconducting Single-Walled Carbon Nanotubes. Spectrosc..

[164] Kharlamova M.V., Kramberger C., Domanov O., Mittelberger A., Yanagi K., Pichler T., Eder D. (2018). Fermi level engineering of metallicity-sorted metallic single-walled carbon nanotubes by encapsulation of few-atom-thick crystals of silver chloride. J. Mater. Sci..

[165] Kharlamova M.V., Kramberger C., Domanov O., Mittelberger A., Saito T., Yanagi K., Pichler T., Eder D. (2018). Comparison of Doping Levels of Single-Walled Carbon Nanotubes Synthesized by Arc-Discharge and Chemical Vapor Deposition Methods by Encapsulated Silver Chloride. Phys. Status Solidi (b).

[166] Kharlamova M.V. (2013). Comparison of influence of incorporated 3d-, 4d- and 4f metal chlorides on electronic properties of single-walled carbon nanotubes. Appl. Phys. A-Mer..

[167] Kharlamova M.V., Yashina L.V., Eliseev A.A., Volykhov A.A., Neudachina V.S., Brzhezinskaya M.M., Zyubina T.S., Lukashin A.V., Tretyakov Y.D. (2012). Single-walled carbon nanotubes filled with nickel halogenides: Atomic structure and doping effect. Phys. Status Solidi (b).

[168] Kharlamova M.V., Eliseev A.A., Yashina L.V., Lukashin A.V., Tretyakov Y.D. (2012). Synthesis of nanocomposites on basis of single-walled carbon nanotubes intercalated by manganese halogenides. J. Phys. Conf. Ser..

[169] Kharlamova M.V., Yashina L.V., Volykhov A.A., Niu J.J., Neudachina V.S., Brzhezinskaya M.M., Zyubina T.S., Belogorokhov A.I., Eliseev A.A. (2012). Acceptor doping of single-walled carbon nanotubes by encapsulation of zinc halogenides. Eur. Phys. J. B.

[170] Kharlamova M.V., Brzhezinskaya M., Vinogradov A., Suzdalev I., Maksimov Y.V., Imshennik V., Novichikhin S.V., Krestinin A.V., Yashina L.V., Lukashin A.V. (2009). The forming and properties of one-dimensional FeHaI 2 (HaI=Cl, Br, I) nanocrystals in channels of single-walled carbon nanotubes. Rus. Nanotechnol..

[171] Sloan J., Friedrichs S., Flahaut E., Brown G., Bailey S.R., Coleman K.S., Xu C., Green M.L.H., Hutchison J.L., Kirkland A.I. (2001). The characterisation of sub-nanometer scale structures within single walled carbon nanotubes. Am. Inst. Phys..

[172] Fedoseeva Y.V., Orekhov A.S., Chekhova G.N., Koroteev V.O., Kanygin M.A., Seovskiy B.V., Chuvilin A., Pontiroli D., Ricco M., Bulusheva L.G. (2017). Single-Walled Carbon Nanotube Reactor for Redox Transformation of Mercury Dichloride. Acs Nano.

[173] Kharlamova M.V., Yashina L.V., Lukashin A.V. (2013). Charge transfer in single-walled carbon nanotubes filled with cadmium halogenides. J. Mater. Sci..

[174] Kharlamova M.V. (2013). Nanoscomposites on Basis of Carbon Nnaotubes: Synthsis, and Modification of the Electronic Properties. Ph.D. Thesis.

[175] Kharlamova M.V. (2016). Electronic properties of single-walled carbon nanotubes filled with manganese halogenides. Appl. Phys. A.

[176] Kharlamova M.V., Kramberger C., Pichler T. (2016). Semiconducting response in single-walled carbon nanotubes filled with cadmium chloride. Phys. Status Solidi (b).

[177] Kharlamova M.V., Kramberger C., Rudatis P., Pichler T., Eder D. (2019). Revealing the doping effect of encapsulated lead halogenides on single-walled carbon nanotubes. Appl. Phys. A.

[178] Kitaura R., Ogawa D., Kobayashi K., Saito T., Ohshima S., Nakamura T., Yoshikawa H., Awaga K., Shinohara H. (2008). High Yield Synthesis and Characterization of the Structural and Magnetic Properties of Crystalline ErCl_3_ Nanowires in Single-Walled Carbon Nanotube Templates. Nano Res..

[179] Satishkumar B.C., Taubert A., Luzzi D.E. (2003). Filling single-wall carbon nanotubes with d- and f-metal chloride and metal nanowires. J. Nanosci. Nanotechnol..

[180] Xu C.G., Sloan J., Brown G., Bailey S., Williams V.C., Friedrichs S., Coleman K.S., Flahaut E., Hutchison J.L., Dunin-Borkowski R.E. (2000). 1D lanthanide halide crystals inserted into single-walled carbon nanotubes. Chem. Commun..

[181] Kharlamova M.V. (2014). Rare-earth metal halogenide encapsulation-induced modifications in Raman spectra of single-walled carbon nanotubes. Appl. Phys. A.

[182] Kharlamova M.V., Volykhov A.A., Yashina L.V., Egorov A.V., Lukashin A.V. (2015). Experimental and theoretical studies on the electronic properties of praseodymium chloride-filled single-walled carbon nanotubes. J. Mater. Sci..

[183] Kharlamova M.V., Kramberger C., Mittelberger A. (2017). Raman spectroscopy study of the doping effect of the encapsulated terbium halogenides on single-walled carbon nanotubes. Appl. Phys. A.

[184] Santidrian A., Kierkowicz M., Pach E., Darvasiova D., Ballesteros B., Tobias G., Kalbac M. (2020). Charge transfer in steam purified arc discharge single walled carbon nanotubes filled with lutetium halides. Phys. Chem. Chem. Phys..

[185] Brown G., Bailey S.R., Sloan J., Xu C.G., Friedrichs S., Flahaut E., Coleman K.S., Hutchison J.L., Dunin-Borkowski R.E., Green M.L.H. (2001). Electron beam induced in situ clusterisation of 1D ZrCl_4_ chains within single-walled carbon nanotubes. Chem. Commun..

[186] Brown G., Bailey S.R., Novotny M., Carter R., Flahaut E., Coleman K.S., Hutchison J.L., Green M.L.H., Sloan J. (2003). High yield incorporation and washing properties of halides incorporated into single walled carbon nanotubes. Appl. Phys. A.

[187] Kirkland A.I., Meyer M.R., Sloan J., Hutchison J.L. (2005). Structure determination of atomically controlled crystal architectures grown within single wall carbon nanotubes. Microsc. Microanal..

[188] Sloan J., Friedrichs S., Meyer R.R., Kirkland A.I., Hutchison J.L., Green M.L.H. (2002). Structural changes induced in nanocrystals of binary compounds confined within single walled carbon nanotubes: A brief review. Inorg. Chim. Acta..

[189] Sloan J., Kirkland A.I., Hutchison J.L., Green M.L.H. (2003). Aspects of crystal growth within carbon nanotubes. Comptes Rendus Phys..

[190] Kharlamova M.V., Eliseev A.A., Yashina L.V., Petukhov D.I., Liu C.P., Wang C.Y., Semenenko D.A., Belogorokhov A.I. (2010). Study of the electronic structure of single-walled carbon nanotubes filled with cobalt bromide. JETP Lett..

[191] Kharlamova M.V. (2015). Raman Spectroscopy Study of the Doping Effect of the Encapsulated Iron, Cobalt, and Nickel Bromides on Single-Walled Carbon Nanotubes. J. Spectrosc..

[192] Bendall J.S., Ilie A., Welland M.E., Sloan J., Green M.L.H. (2006). Thermal stability and reactivity of metal halide filled single-walled carbon nanotubes. J. Phys. Chem. B.

[193] Chernysheva M.V., Eliseev A.A., Lukashin A.V., Tretyakov Y.D., Savilov S.V., Kiselev N.A., Zhigalina O.M., Kumskov A.S., Krestinin A.V., Hutchison J.L. (2007). Filling of single-walled carbon nanotubes by Cul nanocrystals via capillary technique. Physica E.

[194] Hutchison J.L., Sloan J., Kirkland A.I., Green M.L.H., Green M.L.H. (2004). Growing and characterizing one-dimensional crystals within single-walled carbon nanotubes. J. Electron Microsc..

[195] Kiselev N.A., Zakalyukin R.M., Zhigalina O.M., Grobert N., Kumskov A.S., Grigoriev Y.V., Chernysheva M.V., Eliseev A.A., Krestinin A.V., Tretyakov Y.D. (2008). The structure of 1D CuI crystals inside SWNTs. J. Microsc..

[196] Kiselev N.A., Kumskov A.S., Zakalyukin R.M., Vasiliev A.L., Chernisheva M.V., Eliseev A.A., Krestinin A.V., Freitag B., Hutchison J.L. (2012). The structure of nanocomposite 1D cationic conductor crystal@SWNT. J. Microsc..

[197] Kumskov A.S., Zhigalina V.G., Chuvilin A.L., Verbitskiy N.I., Ryabenko A.G., Zaytsev D.D., Eliseev A.A., Kiselev N.A. (2012). The structure of 1D and 3D CuI nanocrystals grown within 1.5-2.5 nm single wall carbon nanotubes obtained by catalyzed chemical vapor deposition. Carbon.

[198] Meyer R.R., Sloan J., Dunin-Borkowski R.E., Kirkland A.I., Novotny M.C., Bailey S.R., Hutchison J.L., Green M.L.H. (2000). Discrete atom imaging of one-dimensional crystals formed within single-walled carbon nanotubes. Science.

[199] Sloan J., Novotny M.C., Bailey S.R., Brown G., Xu C., Williams V.C., Friedrichs S., Flahaut E., Callender R.L., York A.P.E. (2000). Two layer 4:4 co-ordinated KI crystals grown within single walled carbon nanotubes. Chem. Phys. Lett..

[200] Kharlamova M.V., Kramberger C., Rudatis P., Yanagi K., Eder D. (2019). Characterization of the Electronic Properties of Single-Walled Carbon Nanotubes Filled with an Electron Donor-Rubidium Iodide: Multifrequency Raman and X-ray Photoelectron Spectroscopy Studies. Phys. Status Solidi (b).

[201] Flahaut E., Sloan J., Friedrichs S., Kirkland A.I., Coleman K.S., Williams V.C., Hanson N., Hutchison J.L., Green M.L.H. (2006). Crystallization of 2H and 4H PbI_2_ in carbon nanotubes of varying diameters and morphologies. Chem. Mater..

[202] Philp E., Sloan J., Kirkland A.I., Meyer R.R., Friedrichs S., Hutchison J.L., Green M.L.H. (2003). An encapsulated helical one-dimensional cobalt iodide nanostructure. Nat. Mater..

[203] Sloan J., Grosvenor S.J., Friedrichs S., Kirkland A.I., Hutchison J.L., Green M.L.H. (2002). A one-dimensional BaI_2_ chain with five- and six-coordination, formed within a single-walled carbon nanotube. Aew. Chem. Int. Edit..

[204] Friedrichs S., Falke U., Green M.L.H. (2005). Phase separation of Lal_3_ inside single-walled carbon nanotubes. Chemphyschem.

[205] Friedrichs S., Kirkland A.I., Meyer R.R., Sloan J., Green M.L.H. (2005). LaI_2_@(18,3)SWNT: The unprecedented structure of a LaI_2_ "Crystal," encapsulated within a single-walled carbon nanotube. Microsc. Microanal..

[206] Sloan J., Terrones M., Nufer S., Friedrichs S., Bailey S.R., Woo H.G., Ruhle M., Hutchison J.L., Green M.L.H. (2002). Metastable one-dimensional AgCl1-xIx solid-solution wurzite "tunnel" crystals formed within single-walled carbon nanotubes. J. Am. Chem. Soc..

[207] Falaleev N.S., Kumskov A.S., Zhigalina V.G., Verbitskiy I.I., Vasiliev A.L., Makarova A.A., Vyalikh D.V., Kiselev N.A., Eliseev A.A. (2017). Capsulate structure effect on SWNTs doping in RbxAg1-xI@SWNT composites. Crystengcomm.

[208] Kharlamova M.V. (2014). Comparative analysis of electronic properties of tin, gallium, and bismuth chalcogenide-filled single-walled carbon nanotubes. J. Mater. Sci..

[209] Eliseev A.A., Chernysheva M.V., Verbitskii N.I., Kiseleva E.A., Lukashin A.V., Tretyakov Y.D., Kiselev N.A., Zhigalina O.M., Zakalyukin R.M., Vasiliev A.L. (2009). Chemical Reactions within Single-Walled Carbon Nanotube Channels. Chem. Mater..

[210] Wang Z.Y., Li H., Liu Z., Shi Z.J., Lu J., Suenaga K., Joung S.K., Okazaki T., Gu Z.N., Zhou J. (2010). Mixed Low-Dimensional Nanomaterial: 2D Ultranarrow MoS_2_ Inorganic Nanoribbons Encapsulated in Quasi-1D Carbon Nanotubes. J. Am. Chem. Soc..

[211] Kharlamova M.V., Yashina L.V., Lukashin A.V. (2013). Comparison of modification of electronic properties of single-walled carbon nanotubes filled with metal halogenide, chalcogenide, and pure metal. Appl. Phys. A-Mer..

[212] Kharlamova M.V. (2013). Novel approach to tailoring the electronic properties of single-walled carbon nanotubes by the encapsulation of high-melting gallium selenide using a single-step process. JETP Lett..

[213] Carter R., Sloan J., Kirkland A.I., Meyer R.R., Lindan P.J.D., Lin G., Green M.L.H., Vlandas A., Hutchison J.L., Harding J. (2006). Correlation of structural and electronic properties in a new low-dimensional form of mercury telluride. Phys. Rev. Lett..

[214] Sloan J., Carter R., Meyer R.R., Vlandas A., Kirkland A.I., Lindan P.J.D., Lin G., Harding J., Hutchison J.L. (2006). Structural correlation of band-gap modifications induced in mercury telluride by dimensional constraint in single walled carbon nanotubes. Phys. Status Solidi (b).

[215] Monthioux M. (2012). Introduction to the Meta-Nanotube Book. Carbon Meta-Nanotubes: Synthesis, Properties, and Applications.

[216] Sloan J., Monthioux M. (2012). Filled Carbon Nanotubes: (X@CNTs). Carbon Meta-Nanotubes: Synthesis, Properties, and Applications.

[217] Simon F., Monthioux M. (2012). Fullerenes inside Carbon Nanotubes: The Peapods. Carbon Meta-Nanotubes: Synthesis, Properties, and Applications.

[218] Pfeiffer R., Holzweber M., Peterlik H., Kuzmany H., Liu Z., Suenaga K., Kataura H. (2007). Dynamics of Carbon Nanotube Growth from Fullerenes. Nano Lett..

[219] Kharlamova M.V. (2017). Investigation of growth dynamics of carbon nanotubes. Beilstein J. Nanotechnol..

[220] Kharlamova M.V., Kramberger C. (2021). Metal Cluster Size-Dependent Activation Energies of Growth of Single-Chirality Single-Walled Carbon Nanotubes inside Metallocene-Filled Single-Walled Carbon Nanotubes. Nanomaterials.

